# Interferon-**λ** inhibits immunopathology and boosts T cell–mediated immunity in a mouse model of mucosal viral infection

**DOI:** 10.1172/JCI198863

**Published:** 2026-10-15

**Authors:** Emily Feng, Alexa Mansour, Elizabeth Balint, Alyssa Mark, Sofia Tramonte, Jonathan K. Monteiro, Madison Read, Marie Joe Adaimy, Zhou Xing, Matthias Mack, Sergei V. Kotenko, Deanna M. Santer, Ali A. Ashkar

**Affiliations:** 1 McMaster Immunology Research Centre, Department of Medicine and; 2 Michael G. DeGroote Institute for Infectious Disease Research, McMaster University, Hamilton, Ontario, Canada.; 3 University Hospital Regensburg, Department of Nephrology, Regensburg, Germany.; 4 Department of Microbiology, Biochemistry, and Molecular Genetics, Rutgers New Jersey Medical School, Newark, New Jersey, USA.; 5 Department of Immunology, University of Manitoba, Winnipeg, Manitoba, Canada.; 6 Centre for Discovery in Cancer Research, McMaster University, Hamilton, Ontario, Canada.

**Keywords:** Immunology, Infectious disease, Inflammation, Adaptive immunity, Cytokines, Innate immunity

## Abstract

Interferon lambda (IFN-λ) has emerged as a critical inducer of immune regulation in addition to its mucosal antiviral properties. However, the cellular and molecular mechanisms that govern its dual role during viral infections remain unclear. Here, we discovered that mucosal delivery of IFN-λ abrogates the herpes simplex virus type 2–induced (HSV-2-induced) vaginal tissue pathology observed in the absence of type I IFN signaling, through drastically inhibiting IL-6 production. At the same time, we demonstrated that IFN-λ rescued local production of CD4^+^ T cell–dependent IFN-γ, which is essential for protection against the development of toxic megacolon following genital HSV-2 infection. Mechanistically, we found that IFN-λ enhances the differentiation of CCR2^+^Ly6C^hi^ cells into monocyte-derived antigen-presenting cells, thereby boosting T cell responses. In vivo depletion of CCR2^+^ cells abrogated this phenomenon. Together, these findings suggest that IFN-λ orchestrates a potent antiviral response, inhibiting hyperinflammatory responses while selectively augmenting effector T cell function. This study positions IFN-λ as a promising therapeutic candidate for enhancing local antiviral immunity and offers new insights into the regulation of immune responses in the context of mucosal viral infections.

## Abstract 2

Interferon-λ treatment abrogates viral-induced tissue damage through suppressing IL-6 production, while simultaneously promoting CD4+ T cell responses via CCR2+ monocytes to protect against lethal viral dissemination. These findings position IFN-λ as a multifunctional antiviral that limits inflammation and boosts antiviral immunity.

## Introduction

Mucosal surfaces serve as a vital barrier between the host and the external environment and are often targeted as the primary entry point for many viruses. Tightly regulated mucosal immune responses during viral infection at these barriers are critical for host fitness, as dysregulation can lead to insufficient antiviral responses or immune-mediated tissue damage. Interferons (IFNs), including type I and III IFNs, play an essential role in mediating an antiviral response during various viral infections, such as influenza infection, Zika infection, and rotavirus infection, by inducing the expression of IFN-stimulated genes (1–3). In particular, type III IFNs, also known as IFN-λ, have attracted substantial interest for their potential role in immune regulation at mucosal surfaces due to their rapid production following viral infection and the high expression of the receptor, IFN-λR, on mucosal epithelial cells (4, 5).

Excessive cytokine production during viral infection can drive pathogenic outcomes, such as tissue damage, underscoring the need for therapeutics that can counteract inflammatory pathways without hindering the development of protective immunity. Type I–IFN therapy is often unable to inhibit inflammation and can potentiate hyperinflammatory responses, worsening disease outcomes (6, 7). In contrast, IFN-λ therapy inhibits viral replication and promotes viral clearance without triggering or exacerbating hyperinflammatory responses (6, 8, 9). Although IFN-λ signals through a similar JAK/STAT signaling cascade as type I IFNs, this reduced inflammatory potential is likely due to restricted receptor expression and an inability to induce IRF1 expression (4, 10–13). While the antiviral effects of IFN-λ at mucosal sites are well documented, its broader functions in regulating inflammatory immune responses are less studied (1, 4, 14–16). IFN-λ has been shown to possess antiinflammatory functions, such as limiting neutrophil-mediated tissue damage in murine models of inflammation in the lung, intestinal tract, and skin (1, 17, 18). Furthermore, dysregulated IFN-λ responses have been correlated with hyperinflammation in COVID-19 patients, suggesting a critical role in immune regulation (19, 20).

Here, we use a model of herpes simplex virus type 2 (HSV-2) in the female reproductive tract to understand the immunoregulatory functions of IFN-λ at mucosal sites. Previous studies have shown that IFN-λ, largely produced by dendritic cells (DCs) in the genital mucosa, confers protection in murine models of HSV-2 and Zika virus infections through NF-κB–mediated antiviral signaling (2, 21, 22). IFN-λR expression is observed in a subset of immune cells in mice, such as DCs and neutrophils, and IFN-λ signaling can regulate chemokine expression; however, its role in the genital tract in modulating inflammatory pathways during viral infection remains unclear (1, 17, 18, 23–25). Type I IFNs have been well demonstrated to play a critical role during viral infection in regulating NK cell activity, monocyte migration, and DC function (26, 27). However, how type III IFNs can shape mucosal immune responses requires further investigation. We hypothesize that IFN-λ therapy functions as both a modulator of antiviral immunity and inflammation.

We explore how IFN-λ regulates inflammation and boosts protective immune responses against viral infection in *Ifnar^–/–^* mice that lack the type I–IFN receptor. *Ifnar^–/–^* mice serve as a model of hyperinflammation and high susceptibility to viral infection (28). We found that IFN-λ treatment protected against immune-mediated pathology by suppressing IL-6 production. Additionally, we found that IFN-λ prevented lethal viral dissemination and promoted viral clearance by enhancing CD4^+^ T cell–dependent production of IFN-γ. This induction of IFN-γ production relied on CCR2^+^Ly6C^hi^ monocytes that potentiated CD4^+^ T cell function at the site of infection. These results demonstrate that IFN-λ not only reduces viral replication but modulates local mucosal immune responses, offering insight into its potential as a therapeutic for genital tract and other mucosal site viral infections.

## Results

### IFN-λ treatment inhibits tissue pathology and viral dissemination and promotes viral clearance.

IFN-λ is a powerful player in antiviral defence on mucosal surfaces, but its broader immunomodulatory roles remain underexplored. We sought to investigate whether therapeutic mucosal delivery of IFN-λ could inhibit inflammation without compromising the development of protective immune responses in the genital mucosa following intravaginal HSV-2 strain 333 infection. To investigate the functions of IFN-λ, we administered recombinant IFN-λ2 intravaginally 1 day postinfection (DPI) in *Ifnar^–/–^* mice that lack type I–IFN signaling but produce IFN-λ2/3 at levels comparable to WT mice (Figure 1A and Supplemental Figure 1A; supplemental material available online with this article; https://doi.org/10.1172/JCI198863DS1). *Ifnar^–/–^* mice serve as a model for virus-induced hyperinflammation, as they develop severe vaginal tissue pathology 3 DPI due to elevated IL-6 production (24). IFN-λ–treated mice showed reduced viral loads and increased survival compared with control-treated mice, confirming that IFN-λ can confer protection against HSV-2 infection in the absence of type I–IFN signaling (Figure 1, B and C). Strikingly, administration of IFN-λ treatment markedly reduced vaginal tissue pathology in *Ifnar^–/–^* mice 3 DPI (Figure 1, D–F). This correlated with reduced viral titers at 3 DPI and a near-complete abrogation of IL-6 production (Figure 1, G and H). We further assessed whether another subtype, IFN-λ3, would have a similar effect. IFN-λ3–treated mice similarly showed reduced viral titers and IL-6 levels (Supplemental Figure 1, B and C).

We further investigated the role of IFN-λ in inhibiting IL-6–mediated pathology by infecting mice deficient in IFN-λR (*Ifnlr1^–/–^*) with HSV-2. *Ifnlr1^–/–^* mice exhibited increased epithelial damage, viral titers, and IL-6, although to a lesser extent compared to *Ifnar^–/–^* mice, confirming that IFN-λ signaling suppresses IL-6 production (Supplemental Figure 2, A–E). Overall, these findings demonstrate that type III IFNs possess both antiviral and tissue-protective effects in the genital mucosa.

We have previously demonstrated that NK cell–derived IFN-γ can attenuate IL-6–driven pathology (29). As *Ifnar^–/–^* mice have deficient NK-cell–derived IFN-γ production at 2 DPI, we investigated whether IFN-λ administration could reduce tissue pathology by regulating NK cell function (27, 30). However, IFN-λ treatment did not induce IFN-γ production at 2 DPI compared with PBS-treated mice, and *Ifnlr1^–/–^* mice were not deficient in IFN-γ production compared with WT mice (Figure 1I and Supplemental Figure 2F). These results suggest that IFN-λ therapy can attenuate cytokine-driven tissue pathology independently of regulating NK cell responses.

HSV-2 genital infection causes lethal toxic megacolon through viral spread to the enteric nervous system (31). We next assessed whether IFN-λ treatment could inhibit this viral dissemination. IFN-λ treatment reduced the incidence of toxic megacolon at 5 DPI in *Ifnar^–/–^* mice (Figure 1J). This correlated with a trend toward reduced viral loads in the thoracic-lumbar spine and significantly reduced viral loads in the distal colon and vaginal tissue (Figure 1, K–M), suggesting that IFN-λ treatment inhibits not only local viral replication, but also viral spread. To model whether IFN-λ can directly induce antiviral signaling in neurons, we infected primary mouse cortical neurons derived from WT mice with HSV-2. Consistent with previous studies (32), we found that IFN-λ could not inhibit HSV-2 replication in primary mouse cortical neurons in vitro compared with IFN-β (Figure 1N). Thus, IFN-λ protects against neurodissemination independently of antiviral signaling in neurons.

To further assess the role of type III IFNs in inhibiting neurodissemination, WT and *Ifnlr1^–/–^* mice were infected with low and high doses of HSV-2. At a low inoculum of 100 PFU, *Ifnlr1^–/–^* mice were more susceptible to HSV-2 infection than WT mice (Supplemental Figure 2G). This difference in survival correlated with the ability to successfully establish infection, as no infectious virus was detected in the vaginal washes from infected WT mice, whereas virus was detected in *Ifnlr1^–/–^* mice (Supplemental Figure 2H). At a higher viral dose, *Ifnlr1^–/–^* mice had significantly higher viral titers in the vaginal washes only at 1 DPI, but not at later time points of infection (Supplemental Figure 2I). Additionally, we observed a trend toward increased viral titers in the spine, distal colon, and whole vaginal tissue in *Ifnlr1^–/–^* mice compared with WT mice (Supplemental Figure 2, J–L). These findings suggest that endogenously produced IFN-λ plays a critical role in inhibiting the establishment of viral infection and limiting early viral replication, but is less important in limiting viral spread at later time points of infection. Given the contribution of neurodissemination to disease mortality during HSV-2 infection, we sought to determine whether the tissue-protective effects of IFN-λ could be observed during a non-neurotropic mucosal infection.

Infection with thymidine kinase knockout HSV-2 (HSV-2 TK^–^) typically results in an asymptomatic infection in immunocompetent mice due to its inability to replicate in neuronal cells (33, 34). To assess the protective functions of IFN-λ in the absence of a neurotropic infection, we administered IFN-λ during HSV-2 TK^–^ infection (Figure 1O). Although infectious virus was cleared in the vaginal washes by 7 DPI, *Ifnar^–/–^* mice developed severe genital lesions following HSV-2 TK^–^ infection 3–4 weeks after infection, which was prevented by IFN-λ treatment (Figure 1, P–R). These findings suggest that *Ifnar^–/–^* mice exhibit defective viral clearance and that the protective effects of IFN-λ include not only preventing neurodissemination but also enhancing viral clearance.

### The absence of type I–IFN signaling impairs local T cell immunity.

CD4^+^ T cells are known to be the primary source of IFN-γ at later time points of HSV-2 infection, where their production of IFN-γ contributes to viral clearance, prevents infection of sensory neurons that innervate the vaginal mucosa, and promotes the recruitment of CD8^+^ T cells (26, 35–38). Although type I–IFN signaling is well known to be critical for NK cell–derived IFN-γ production, it is not clear whether it is also necessary for CD4^+^ T cell–dependent IFN-γ production. We evaluated whether *Ifnar^–/–^* mice had any deficiencies in their CD4^+^ T cell responses to HSV-2 infection that contributed to their greater susceptibility to HSV-2 infection compared with WT mice (Figure 2A). We observed that *Ifnar^–/–^* mice had higher viral titers starting 3 DPI and produced no IFN-γ at 5 DPI compared with WT mice (Figure 2, B and C). In contrast, *Ifnlr1^–/–^* mice produced similar levels of IFN-γ compared with WT mice (Supplemental Figure 2M). The lack of IFN-γ in *Ifnar^–/–^* mice correlated with a significant reduction in the recruitment of T cells, including CD4^+^, CD8^+^, and TCRγδ^+^ T cells, but not total immune cells to the vaginal tissue during HSV-2 infection compared with WT mice (Figure 2, D–H). Furthermore, we observed that the impaired recruitment of T cells correlated with reduced production of CXCL9 and CXCL10 in *Ifnar^–/–^* mice at 2 and 3 DPI (Figure 2, I and J). These results suggest that the absence of type I–IFN signaling impairs lymphocyte recruitment during genital HSV-2 infection.

### IFN-λ treatment boosts lymphocyte function.

Although IFN-λR expression is limited in immune cell populations in mice, we hypothesized that IFN-λ treatment could protect against viral dissemination and promote viral clearance by increasing chemokine production and T cell recruitment in *Ifnar^–/–^* mice (Figure 3A). Strikingly, treatment with either IFN-λ2 or IFN-λ3 potently rescued IFN-γ production at 5 DPI (Figure 3B and Supplemental Figure 3A). Since IFN-λ treatment abrogated IL-6 production, we assessed whether this loss of IL-6 was related to the rescue in IFN-γ production. We administered an α-IL-6 antibody to *Ifnar^–/–^* mice, which depleted IL-6 levels 3 DPI (Supplemental Figure 3B). Depletion of IL-6 did not rescue IFN-γ production like IFN-λ treatment (Supplemental Figure 3C). Thus, IFN-λ treatment rescues IFN-γ production through a mechanism distinct from its ability to suppress IL-6 production.

IFN-λ–treated mice had significantly higher levels of chemokines CXCL9 and CXCL10 in vaginal washes compared with PBS-treated mice, suggesting that IFN-λ signaling could increase immune cell recruitment to the genital mucosa (Figure 3, C and D). However, the total number of CD45^+^ leukocytes and CD3^+^ lymphocytes, including CD4^+^, CD8^+^, and TCRγδ^+^ T cells, remained unchanged in the IFN-λ–treated mice compared with control-treated mice (Figure 3, E–I). Others have shown that IFN-λ inhibits neutrophil recruitment in a mouse model of HSV-1 skin lesions by suppressing chemokine production to limit neutrophil-mediated pathology (39). We also observed a reduced number and proportion of Ly6G^+^ neutrophils in the vaginal tract 5 DPI, despite higher CXCL5 production (Supplemental Figure 3, E and F). Taken together, we found that, while IFN-λ potently induces IFN-γ production to limit viral spread, this is independent of increasing T cell recruitment. Thus, it is unclear whether T cells are necessary for IFN-γ production in IFN-λ–treated mice.

### IFN-λ–mediated protection relies on recruited CD4^+^ T cells.

To assess whether T cells are necessary for IFN-γ production induced by IFN-λ treatment, IFN-λ–treated mice received fingolimod (FTY720) to inhibit lymphocyte egress from secondary lymphoid tissues (Figure 3J). FTY720 treatment ablated IFN-γ production in IFN-λ–treated mice and significantly reduced the total number of CD4^+^, CD8^+^, and TCRγδ^+^ T cells, without altering viral titers (Figure 3, L–P). Interestingly, FTY720 treatment alone substantially reduced the incidence of toxic megacolon to rates comparable with mice treated with IFN-λ alone (Figure 3K). This finding suggests that lymphocytes recruited to the gastrointestinal tract may contribute to damage to the peripheral nervous system, similar to what has been reported during West Nile virus infection (40). The combined treatment of FTY720 and IFN-λ in *Ifnar^–/–^* mice did not confer additional protection beyond either treatment alone, suggesting that the protective functions of IFN-λ are dependent on recruited lymphocytes.

To further assess the protective mechanisms of IFN-λ, we administered an anti-CD4 depleting antibody, which effectively depleted CD4^+^ T cells in the draining lymph node (dLN) in both PBS- and IFN-λ–treated mice (Figure 3Q and Supplemental Figure 3G). CD4^+^ T cell depletion alone did not reduce the incidence of toxic megacolon or affect viral titers. However, α-CD4 treatment blunted IFN-γ production in IFN*-*λ–treated mice, and reversed the protective effect of IFN-λ (Figure 3, R–T). This was associated with a reduction in the number of CD4^+^ T cells, but not TCRγδ^+^ or CD8^+^ T cells in the vaginal tract (Figure 3, U–W). Together, these results support a requirement for CD4^+^ T cells, but not TCRγδ^+^ or CD8^+^ T cells, in IFN-λ–dependent IFN-γ production in the vaginal tract following HSV-2 infection.

### IFN-λ does not alter T cell priming in the dLN.

As there were no differences in the total number of T cells in the vaginal tissue, we assessed whether IFN-λ treatment influenced T cell priming in the dLN. Although *Ifnlr1* mRNA is not expressed in murine lymphocytes, it is expressed in murine DCs (23). Previous studies have shown that IFN-λ signaling plays a key role in DC migration and priming of CD8^+^ T cell responses (23). We assessed whether there were differences in DC migration and activation between WT and *Ifnar^–/–^* mice, and whether IFN-λ treatment was able to influence DC activity. *Ifnar^–/–^* mice showed impaired migration of classical DCs (cDCs) to the dLN compared with WT mice; however, this was not altered by IFN-λ treatment (Supplemental Figure 6A and Figure 4A). Additionally, the total number of monocyte-derived DCs (moDCs) was unchanged among WT mice, *Ifnar^–/–^* mice, and *Ifnar^–/–^* mice treated with IFN-λ (Figure 4B). Interestingly, DCs in the dLN from WT mice had higher expression of CD86, but lower expression of CD80 compared with *Ifnar^–/–^* mice (Figure 4, C, E, and F). CD40 expression was mildly lower in *Ifnar^–/–^* mice compared with WT mice (Figure 4, C and D). Furthermore, DCs from WT mice had a distinct MHC II^hi^ population that was absent in *Ifnar^–/–^* mice (Figure 4, C and G). IFN-λ treatment failed to increase the expression of any of these activation markers compared with PBS-treated mice (Figure 4, C–G). Thus, while loss of type I–IFN signaling impaired DC maturation and migration to the dLN, IFN-λ was unable to rescue DC activation. While we did not observe any differences in DC migration or activation in the dLN following IFN-λ treatment, we assessed whether T cell function may still be altered by IFN-λ treatment through other mechanisms. *Ifnar^–/–^* mice had reduced numbers of T cells in the dLN compared with WT mice*,* which was not rescued by IFN-λ treatment (Figure 4, H–K). We further confirmed previous findings that *Ifnar^–/–^* mice do not have a defect in T cell priming, as we observed a significant increase in the proportion of IFN-γ–secreting T cells and total IFN-γ production after ex vivo HSV-2 stimulation of dLN cells from *Ifnar^–/–^* mice compared with WT mice (Figure 4, L, M, and O) (26). We also found that IFN-λ treatment did not increase the proportion of IFN-γ–secreting CD4^+^ or CD8^+^ T cells in *Ifnar^–/–^* mice (Figure 4, L and M). IL-17A^+^ T cells have been demonstrated to be important for optimal TH1 responses (41). However, while there was a slight increase in IL-17A–secreting CD4^+^ T cells in *Ifnar^–/–^* mice compared with WT mice*,* this was unchanged in IFN-λ–treated mice 5 DPI (Figure 4N). Finally, total IFN-γ production in the dLN did not differ between any of the groups (Figure 4O). Overall, our findings confirm that IFN-λ treatment does not improve T cell priming in the dLN or alter DC activation.

### Type II IFN does not rescue impaired lymphocyte-dependent IFN-γ production.

Type I, II, and III IFNs share many overlapping and interconnected functions that influence immune cell function. Given that *Ifnar^–/–^* mice exhibit impaired NK cell–derived IFN-γ production, we investigated whether impaired T cell production of IFN-γ was due to a loss of type I IFN signaling or NK cell–derived IFN-γ production. To model early IFN-γ production by NK cells, we administered recombinant IFN-γ 1.5 and 2 DPI to determine whether early IFN-γ production was necessary or sufficient for proper CD4^+^ T cell responses (Figure 5A). IFN-γ treatment did not prevent the development of megacolon in *Ifnar^–/–^* mice, and only modestly reduced viral titers at 3 DPI (Figure 5, B and C). Additionally, IFN-γ treatment did not rescue IFN-γ production by T cells at 5 DPI or increase the number of T cells in vaginal tissue (Figure 5, D–H). To support these findings, we depleted NK cells in WT mice, which reduced the total number of NK1.1^+^ cells in the vaginal tissue and reduced IFN-γ levels in the vaginal washes 2 DPI without altering local viral loads (Supplemental Figure 4, A–C). However, NK cell depletion did not impair T cell recruitment or T cell–dependent IFN-γ production (Supplemental Figure 4, D–G). Overall, these findings demonstrate that early IFN-γ production is neither necessary nor sufficient for an effective T cell response. Interestingly, IFN-γ treatment significantly increased the number of CD45^+^ cells, including T cells and DCs in the dLN, suggesting that reduced DC migration and T cell accumulation in *Ifnar^–/–^* mice may partially result from impaired NK cell function (Figure 5, I–K). Furthermore, these findings suggest that changes observed in the dLN do not necessarily reflect immune cell function at the site of infection, and that T cell function in the vaginal tissue may be influenced by the local tissue environment.

### IFN-λ–mediated induction of T cell–dependent IFN-γ production relies on CCR2^+^Ly6C^hi^ cells.

As there was no difference in the priming of T cell responses in the dLN following IFN-λ treatment, we assessed whether we could observe differences in T cell function in the vaginal tissue. While we did not observe differences in the proportion of IFN-γ–secreting CD4^+^ or CD8^+^ T cells in the vaginal tract 5 DPI, there was a significant increase in total IFN-γ production following ex vivo stimulation (Figure 6, A–C). These results suggest that IFN-λ treatment increased the overall IFN-γ production per T cell without increasing the frequency of IFN-γ–secreting T cells. Previous studies have shown that moDCs derived from CCR2^+^ monocytes are dispensable for T cell priming during HSV-2 infection, but are critical for restimulating activated T cells to induce IFN-γ production at the site of infection (26). Indeed, we find that WT mice depleted of CCR2^+^ monocytes have deficient IFN-γ production by NK cells 2 DPI, and T cells 5 DPI (Supplemental Figure 5A). Additionally, *Ifnar^–/–^* mice have been shown to have deficient CCR2^+^ monocyte recruitment due to reduced CCR2 ligand production (26, 27). Moreover, we found that *Ifnar^–/–^* mice have reduced numbers of CD11b^+^ DCs in the vaginal tract 5 DPI compared with WT mice (Supplemental Figure 5, B and C, and Supplemental Figure 6B). Interestingly, IFN-λ treatment significantly increased the number of CD11b^+^ DCs in the vaginal tract (Figure 6, D and E), but did not influence their activation, as measured by MHC II, CD40, CD80, and CD86 (Supplemental Figure 5, D–G). Additionally, while we did not observe a significant difference in the total number of monocytes at 5 DPI, there was an increase in the expression of MHC II on both Ly6C^hi^CCR2^+^ and Ly6C^lo^CCR2^–^ monocytes following IFN-λ treatment (Figure 6, F–J). When Ly6C^hi^ monocytes differentiate into moDCs, they upregulate MHC II and CD11c and downregulate Ly6C and CCR2 (42, 43). These findings suggest that, in IFN-λ–treated mice, the upregulation of MHC II on Ly6C^hi^ cells and Ly6C^lo^ monocytes indicated an increase in differentiation into CD11b^+^ moDCs (Figure 6K).

To assess whether CCR2^+^ monocytes and moDCs were required for IFN-λ–induced IFN-γ production, IFN-λ–treated *Ifnar^–/–^* mice were administered PBS or α-CCR2 (Figure 6L). α-CCR2 treatment did not reduce local viral titers, suggesting that recruitment of CCR2^+^ monocytes does not play a role in controlling local viral replication (Figure 6M). Depletion of CCR2^+^ cells completely abolished the induction of IFN-γ, without reducing CD4^+^ or CD8^+^ T cell recruitment (Figure 6, N–P). Additionally, depletion of CCR2^+^ cells reduced the total number of Ly6C^hi^CCR2^+^ monocytes, Ly6C^lo^CCR2^–^ monocytes, and CD11b^+^ DCs, suggesting that Ly6C^lo^CCR2^–^ monocytes and CD11b^+^ DCs were derived from Ly6C^hi^CCR2^+^ monocytes (Figure 6, Q–S). Overall, these findings indicate that IFN-λ relies on CCR2^+^ monocytes to rescue CD4^+^ T cell–dependent IFN-γ production.

## Discussion

In this study, we investigated the function of IFN-λ in modulating immune responses in mucosal tissues during HSV-2 viral infection. Using *Ifnar^–/–^* mice, which lack type I–IFN signaling and exhibit impaired early type II IFN production, we demonstrate that IFN-λ simultaneously suppresses immune-mediated pathology while also enhancing antiviral CD4^+^ T cell responses. These findings reveal a triple function for IFN-λ in inducing direct antiviral signaling, regulating hyperinflammatory responses, and inducing protective innate and adaptive immune responses to optimize host fitness during viral infection.

Control of viral replication is not always sufficient to prevent virus-induced pathology. While type I and type III IFNs share similar antiviral signaling during viral infection, their roles in regulating inflammation differ. Type I IFN signaling can either potentiate or inhibit cytokine-driven pathology, but the regulatory function of IFN-λ and its similarities or differences to that of type I IFNs have not been widely examined (28, 44). In this study, we found that IFN-λ significantly dampens IL-6 production, thereby limiting virus-induced pathology in *Ifnar^–/–^* mice. These findings align with previous studies that demonstrate that IFN-λ possesses antiinflammatory properties, inhibiting immune-mediated pathology following infection (1, 17, 18, 25, 45). While the early timing of type I IFN therapy is typically necessary for protection against viral infection, the protective effects of IFN-λ appear to be less time sensitive (6, 46, 47). We found that IFN-λ administration at 1 DPI protected against virus-induced pathology. Additionally, the prolonged production of IFN-λ compared with type I IFNs during viral infection suggests its unique role in mitigating hyperinflammatory responses over an extended time frame (1, 11, 47). That being said, prolonged IFN-λ signaling has also been shown to impair epithelial repair, as seen in the lung and gut mucosa (48–50). Further research is needed to explore how IFN-λ interacts with specific immune cells to modulate cytokine production and inflammation at different phases of viral infection.

CD4^+^ T cells are crucial for mounting a neuroprotective immune response during HSV-2 infection through the production of IFN-γ (37, 51, 52). Not only does IFN-γ signaling induce expression of nitric oxide to inhibit viral replication and viral spread, but CD4^+^ T cell–derived IFN-γ is also necessary for CXCL9 and CXCL10 production and subsequent recruitment of other neuroprotective CXCR3^+^ lymphocytes (35, 38, 53). In our study, we demonstrate that the absence of type I IFN signaling impairs CD4^+^ T cell responses following HSV-2 infection, resulting in increased viral spread to the spine and distal colon, and a high incidence of toxic megacolon. We find that impaired CD4^+^ T cell recruitment was associated with a lack of CXCL9 and CXCL10 production. Moreover, loss of type I IFN–induced CCR2 ligand expression disrupts the recruitment of monocyte-derived DCs that stimulate IFN-γ production by CD4^+^ T cells (26). These CD4^+^ T cell responses may also be necessary for efficient viral clearance from the vaginal tract, as *Ifnar^–/–^* mice developed genital lesions following HSV-2 TK^–^ infection despite no detectable virus in the vaginal washes. HSV-2 TK^–^ inoculation typically induces the rapid production of neutralizing IgG2c, which supports viral clearance and prevents viral infection of sensory neurons (54–56). However, these protective antibody responses are dependent on CD4^+^ T cells and IFN-γ (56, 57). Thus, our findings highlight the importance of type I IFNs in generating protective adaptive immune responses during mucosal viral infection. However, we also find that lymphocytes are likely involved in pathogenic immune responses in the ENS, leading to gut dysmotility. Recently, antigen-specific T cell responses have been described to induce damage to neuronal and glial cells in the ENS following WNV infection (40). Antigen-nonspecific T cell responses have also been shown to induce neuronal cell death following ZIKV infection (58). Further exploration is needed into the potential role of type I and III IFNs in regulating pathogenic T cell responses.

Given that T cells are predominant producers of IFN-γ during mucosal viral infection, our data suggest that IFN-λ is a potent inducer of CD4^+^ T cell–dependent production of IFN-γ, uncovering a previously unrecognized role of IFN-λ in orchestrating adaptive immune responses at mucosal sites and promoting viral clearance and protecting against viral spread. The lack of detectable *Ifnlr1* expression in murine CD4^+^ T cells suggests an indirect role of IFN-λ in potentiating adaptive immunity, as previously observed for CD8^+^ T cell function during IAV and SARS-CoV-2 infection (23, 24). Consistent with this, IFN-λ has been demonstrated to enhance NK cell–derived IFN-γ production and TH1 differentiation in allergic asthma models (59–61). Critically, we find that in our genital HSV-2 infection model, *Ifnlr1^–/–^* mice are more susceptible to low doses of viral infection compared with WT mice but have an intact CD4^+^-dependent protective response at higher viral doses. These findings align with prior studies demonstrating that endogenous IFN-λ production provides critical early antiviral defence, particularly at low viral doses, but more robust antiviral adaptive immune responses rely on type I IFNs (1, 62).

Notably, type III IFNs promoted CD4^+^ T cell responses in a mechanism that was distinct from type I IFNs. IFN-λ treatment did not increase T cell recruitment, enhance T cell priming in the draining lymph nodes, or rescue monocyte recruitment. Instead, IFN-λ promoted the accumulation of CD11b^+^ DCs and upregulated MHC II expression in Ly6C^hi^ and Ly6C^lo^ monocytes, suggesting that IFN-λ drives monocyte differentiation into antigen-presenting DCs (42, 43). The precise mechanisms by which IFN-λ affects monocyte differentiation remain unclear, including whether it directs or accelerates monocyte fate or alters the type of monocytes recruited. Prior studies have shown that disparities in PU.1 expression in Ly6C^hi^ monocytes determine monocyte differentiation into either moDCs or iNOS^+^ macrophages (63). We demonstrate that IFN-λ promotes the accumulation of moDCs, which we hypothesize restimulate CD4^+^ T cell IFN-γ production through MHC:TCR interactions (26, 64, 65). In contrast to type I IFNs, IFN-λ selectively induced CD4^+^ T cell–dependent IFN-γ production, but not NK cell–derived IFN-γ, which depends on monocyte production of IL-12 and IL-18 (27, 30, 66). A limitation of our study is that we do not directly assess APC-T cell interactions or isolate IFN-γ production specifically from CD4^+^ cells. Therefore, the observed IFN-γ production may occur through alternative mechanisms or cell types that nevertheless depend on CCR2^+^ monocytes and CD4^+^ T cells. Overall, our findings support the idea that IFN-λ enhances the capacity of monocyte-derived cells to support T cell activity, consistent with previous reports that type III IFN signaling on bone-marrow–derived DCs enhances antigen uptake and T cell activation (23). However, the precise mechanisms require further investigation.

Impaired adaptive immune responses and a propensity for hyperinflammatory immune responses are frequently observed in susceptible populations, including those with immunodeficiencies or advanced age. Aging is associated with reduced IFN responses, which can impact the ability to mount effective adaptive immune responses (67). For example, delayed type I IFN induction in patients with SARS-CoV-2 is associated with more severe disease outcomes (68). However, therapeutic administration of type I IFNs can inadvertently induce inflammation (7). In contrast, clinical trials have shown that a single injection of IFN-λ accelerates viral load decline in COVID-19 patients without major side effects (8, 9). Our data suggest that IFN-λ treatment can also accelerate viral clearance by promoting effective adaptive immune responses without inducing inflammatory cytokine production. Given the critical role of CD4^+^ T cells in establishing resident memory CD8^+^ T cell responses and effective B cell responses that protect against subsequent HSV-2 challenge (38, 57, 69), our findings support the potential of IFN-λ as a vaccine adjuvant aimed at enhancing memory T cell and B cell responses. Overall, our findings highlight the triple function of IFN-λ in inhibiting viral replication, fine-tuning the immune response to limit pathogenic innate immune responses, and potentiating protective adaptive immune responses.

## Methods

### Sex as a biological variable.

Our study exclusively examined female mice because the disease model involves infection of the female reproductive tract.

### Mouse model.

*Ifnar^–/–^* mice were bred onto a C57BL/6J background, and breeding pairs were provided by Laurel Lenz (University of Colorado, Aurora, Colorado, USA). *Ifnlr1^–/–^* mice on a C57BL/6J background were provided by Rutgers New Jersey Medical School. C57BL/6J (WT) mice were purchased from The Jackson Laboratory. Colonies of WT, *Ifnar1^–/–^* and *Ifnlr1^–/–^* mice were bred and maintained at McMaster University Central Animal Facility. Mice were housed under specific pathogen–free conditions in a temperature-controlled environment (21 ± 1°C), on a 12-hour light/12-hour dark cycle, and a maximum of 5 mice per cage. Mice were fed an irradiated Teklad global 18% protein diet (catalog 2918) with ad libitum access to food and water. Mice that were 8–16 weeks old were infected with HSV-2 strain 333 or HSV-2 TK^–^. All mice were age-matched across groups for experiments. Mice were monitored daily for disease severity based on the following scale: 0, no apparent infection; 1, slight redness of the external vagina; 2, redness and swelling of the external vagina and surrounding tissues; 3, severe redness and swelling of the external vagina and surrounding tissues; 4, genital ulceration and severe redness, swelling, and hair loss in the genital and surrounding tissues and external evidence of constipation; 5, severe genital ulceration extending to surrounding tissues and external evidence of megacolon/severe constipation. Mice were euthanized upon reaching a score of 4 or 5 for humane considerations. Megacolon was determined by examination of dissected colons at the study endpoint of 5 DPI. Samples were classified as having megacolon when compacted fecal material and bowel distension were observed in at least 50% of the colon.

### Virus infection and in vivo treatments.

Female mice were injected s.c. with 2 mg DMPA 5 days before HSV-2 infection. Mice were anesthetized intraperitoneally (i.p.) with ketamine/xylazine and infected intravaginally with 10 μL of HSV-2 strain 333 at the indicated PFUs. Vaginal washes were collected by pipetting 30 μL of PBS 5 times in and out of the vaginal canal, repeated twice. A total of 5 μg of mouse rIFN-λ2 (PeproTech), mouse rIFN-λ3 (provided by Warren Wakarchuk, University of Alberta), or 1,000 U IFN-β (R&D) was administered intravaginally after anesthetization with ketamine and xylazine. To administer FTY720, 1 mg/kg/d was administered in the drinking water starting from 5 days before infection. To deplete CD4^+^ T cells, we administered 200 μg α-CD4 (GK1.5) by i.p. injection at –2 and 1 DPI. To deplete NK cells, we administered 200 μg of α-NK1.1 (BioXCell, PK136) at –1, 1, and 3 DPI. To deplete IL-6, 100 μg α-IL-6 (BioXCell, MP5-20F3) was administered by i.p. injection from –1 to 4 DPI. To deplete CCR2^+^ cells, 20 μg of α-CCR2 (MC-21) was administered by i.p. injection from –2 to 4 DPI. To isolate tissues for viral titers, mice were anesthetized with isoflurane and perfused with 10 mL of PBS. Tissues were homogenized with the Bio-Gen Pro200 Homogenizer. Spinal tissue was homogenized in 1 mL of PBS, and vaginal tissue and distal colon were homogenized in 200 μL of PBS.

### Viral titration.

HSV-2 strain 333 and HSV-2 TK^–^ were grown in Vero cells (ATCC), which were maintained in αMEM supplemented with 10 mM HEPES, 2 mM l-glutamine, 100 U/mL penicillin/streptomycin, and 5% fetal bovine serum (FBS). The viral load of cell lysates, vaginal lavages, spine homogenates, whole vaginal tissue homogenates, and distal colon homogenates were detected through plaque assays on Vero cells. Viral dilutions in supplemented α-ΜΕΜ were incubated on Vero cells in 100–200 μL for 1 hour at 37°C, 5% CO_2_ with constant rocking, and then overlaid with 2 mL of fully supplemented 5% αMEM. 48 hours later, cells were fixed and stained with crystal violet, and the plaques were quantified.

### Mouse tissue histology.

The vaginal canals were isolated at 3 DPI and fixed in 2% paraformaldehyde (PFA) for 48 hours and then stored in 70% ethanol. The tissue was embedded in paraffin, cross-sectioned, stained with H&E, and the slides were scanned with Aperio ScanScope XT (Leica Biosystems). Vaginal pathology was scored on a scale of 0 to 6, with separate scores given for epithelium damage and submucosal damage. Epithelial damage was scored from 0 to 3 for no loss, small and localized epithelium loss, 50% epithelium loss, and complete or almost complete loss of epithelium. Submucosal damage was scored from 0 to 3 for no damage, mild damage and hemorrhaging, moderate damage and hemorrhaging, and severe tissue damage and hemorrhaging extending to the entire tissue submucosa. Scores were totalled to give the overall pathology score. Epithelial lining was quantified in Fiji Image J Software.

### Primary cortical neuron cultures.

Primary cortical neurons were grown on plates coated with 0.1 mg/mL Poly-d-Lysine (Gibco) overnight at 37°C. The meninges from the cortices of E17.5 C57BL/6J mice were carefully removed, and the remaining tissue was digested with 0.25% trypsin (Gibco), 1 mg/mL DNase (Sigma) in 1X HBSS (Gibco) for 20 minutes. Cortices were homogenized with a fire-polished pipette and plated in Neurobasal Medium (Gibco) supplemented with 1% FBS, 100 U/mL penicillin/streptomycin**,** 2 mM GlutaMAX (Gibco), and 1X B-27 Supplement (Gibco). At 24 hours later, the media were changed to FBS-free media. Cell culture media were changed every 2–3 days. Primary neurons were stimulated with indicated concentrations of rIFN-λ2 and rIFN-β for 24 hours, then infected with HSV-2 at an MOI of 1. Cell culture supernatants and cell lysates were collected 24 hours after 3 successive freeze-thaws and vigorous vortexing.

### Flow cytometry.

Vaginal tissue single-cell suspensions were isolated through finely mincing the tissue, followed by enzymatic digestion with 0.5 IU of Collagenase A (Roche) for 1 hour at 37°C twice. Lymph node single-cell suspensions were isolated by gently disrupting them against a scratched plate. Live/dead cells were discriminated using eFluor 780 fixable viability dye (eBioscience) for 30 minutes at room temperature, protected from light. The cells were then blocked with anti-CD16/CD32 (eBioscience) for 20 minutes. Cells were subsequently stained for extracellular markers anti-mouse CD45 (eBioscience, 30-F11), anti-mouse CD3 (BD Biosciences, 145-2C11), anti-mouse CD4 (BD Biosciences, GK1.5, eBioscience, RM4-4), anti-mouse CD8 (eBioscience, 53-6.7), anti-mouse TCR-β (BioLegend, H57-597), anti-mouse TCR-γδ (BD Biosciences, GL3), anti-mouse NK1.1 (BioLegend, PK136), anti-mouse B220 (BD Biosciences, RA3-6B2), anti-mouse Ly6G (BD Biosciences, 1A8), anti-mouse Ly6C (BioLegend, HK1.4), anti-mouse CD11b (BD Biosciences, M1/70), anti-mouse CD11c (BD Biosciences, HL3), anti-mouse I-A/I-E (BioLegend, M5/114.15.2), anti-mouse CD64 (BioLegend, X54-5/7.1), anti-mouse CD26 (BD Biosciences, H194-112), anti-mouse CCR2 (SA203G11), anti-mouse CD40 (BD Biosciences, 3/23), anti-mouse CD80 (BioLegend, 16-10A1), anti-mouse CD86 (BioLegend, GL-1) for 30 minutes. Samples were fixed for 20 minutes with 2% PFA. Alternatively, cells were stained for intracellular cytokines using the BD Pharmingen Mouse Intracellular Cytokine Staining Kit anti-mouse IFN-γ (XMG1.2) and anti-mouse IL-17A (TC11-18H10.1). Sample acquisition was carried out using the Cytek Aurora and analyzed using FlowJo software (v10.10.0).

### Ex vivo stimulation.

Lymph node single-cell suspensions were stimulated at a concentration of 5,000 cells/μL with 10 μg/mL of HSV-2 lysate for 24 hours in RPMI supplemented with 10 mM HEPES, 2 mM l-glutamine, 100 U/mL penicillin/streptomycin, 10% FBS, 10 mM sodium pyruvate (Gibco), non-essential amino acids (Gibco), and 50 μM β-mercaptoethanol (Sigma). Total vaginal tissue single-cell suspensions were incubated for 24 hours in 200 μL of RPMI supplemented with 10 mM HEPES, 2 mM l-glutamine, 100 U/mL penicillin/streptomycin, 10% FBS, 10 mM sodium pyruvate (Gibco), non-essential amino acids (Gibco), and 50 μM β-mercaptoethanol (Sigma). GolgiPlug (BD Biosciences) was added to a final concentration of 2 μM during the last 4 hours of incubation. Cell culture supernatants were collected at 24 hours for analysis by ELISA.

### Cytokine detection.

Vaginal lavages diluted in PBS and cell culture supernatants were analyzed for cytokine levels through the Eve Technologies Mouse Cytokine/Chemokine 44-Plex Discovery Assay Array. IFN-λ2/3, CXCL9, CXCL10, IFN-γ, and IL-6 were independently quantified in vaginal lavages using R&D DuoSet ELISA kits.

### Statistics.

Data are presented as mean ± SEM. Statistical differences were analyzed using a 2-tailed Student’s *t* test between 2 groups and a 2-way ANOVA for multigroup comparisons. A Kaplan-Meier survival analysis followed by the Mantel-Cox log-rank test was used for survival curve analysis. Graphs and statistical analysis were completed using GraphPad Prism 10. Statistical significance is indicated as *P < 0.05, **P < 0.01, ***P < 0.001, ****P < 0.0001.

### Study approval.

All animal experiments were approved by the Animal Research Ethics Board at McMaster University.

### Data availability.

All data are available in the main text or supplemental materials. Values for all data points are provided in the Supporting Data Values file.

## Author contributions

Conceptualization: EF and AAA. Methodology: EF and AAA. Investigation: EF, AM, EB, AM, MR, MJA, ST, and JKM. Resources: ZX, SVK, MM, DMS, and AAA. Visualization: EF. Funding acquisition: AAA. Project administration: EF and AAA. Supervision: AAA. Writing – original draft: EF. Writing – review & editing: EF, AM, EB, DMS, and AAA.

## Funding support

- Canadian Institutes of Health Research Project Grant 20009360 (AAA).
- Canadian Institutes of Health Research Graduate Scholarship FBD-181475 (EF).

## Supplementary Material

Supplemental data

Supporting data values

## Figures and Tables

**Figure 1:**
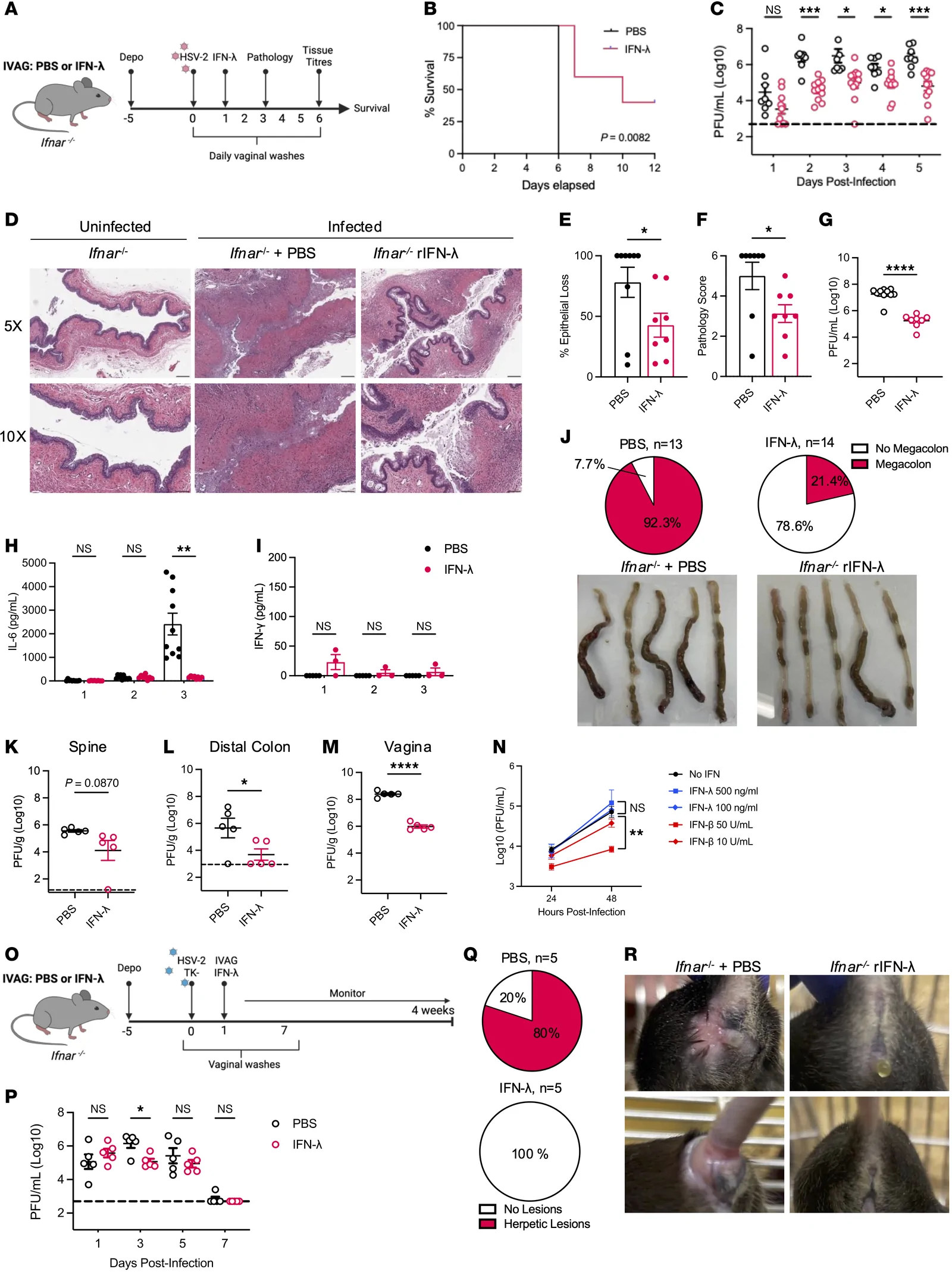
IFN-λ treatment inhibits tissue pathology and viral dissemination and promotes viral clearance. (**A**) *Ifnar^–/–^* mice were infected with 10^3^ PFU HSV-2 333 and administered PBS or IFN-λ (*n =* 8, 11). Data representative of 2 pooled experiments. (**B**) Survival rates between PBS- and IFN-λ–treated mice. (**C**) Viral titers in vaginal washes. (**D**–**J**) *Ifnar^–/–^* mice were infected with 10^4^ PFU HSV-2 strain 333 and administered PBS or IFN-λ. Data representative of 2 independent experiments. (**D**) Representative vaginal histology 3 DPI. Scale bars: 5 ×, 100 μm; 10 ×, 200 μm, respectively. (**E**) Percentage epithelial loss (*n* = 8, 9). (**F**) Pathology score. (**G**) Viral titers in vaginal washes 3 DPI. (**H**) IL-6 levels in vaginal washes (*n* = 10, 9). (**I**) IFN-γ levels in vaginal washes (*n* = 5, 5). mice. (**J**) Representative distal colon images and rates of toxic megacolon 5 DPI, pooled from 3 independent pooled experiments. (**K**–**M**) *Ifnar^–/–^* mice were infected with 10^3^ PFU HSV-2 333 and administered PBS or IFN-λ (*n =* 5, 5). Data representative of 2 independent experiments. Viral titers in the (**K**) spine, (**L**) distal colon, and (**M**) vaginal tissue 6 DPI. (**N**) Primary cortical neurons from WT mice were stimulated with indicated concentrations of IFN-λ2 or IFN-β and infected with HSV-2. Infectious viral loads at the indicated time points in triplicate. (**O**) *Ifnar^–/–^* mice were infected with 10^4^ PFU HSV-2 TK^–^ and administered PBS or IFN-λ. Data shown are representative of 2 independent experiments. (**P**) Viral titers in vaginal washes (*n =* 5, 5). (**Q**) Rates of developing severe genital lesions. (**R**) Representative of genital lesion images. *P < 0.05, **P < 0.01, and ****P < 0.0001. Statistical significance determined by Kaplan-Meier survival analysis (**B**), repeated-measure 2-way ANOVA with Šídák’s multiple comparisons test (**C**, **H**, **I**, and **P**), ordinary 2-way ANOVA with Dunnett’s multiple comparisons test (**N**), and 2-tailed Student’s *t* test (**E**–**G** and **K**–**M**).

**Figure 2:**
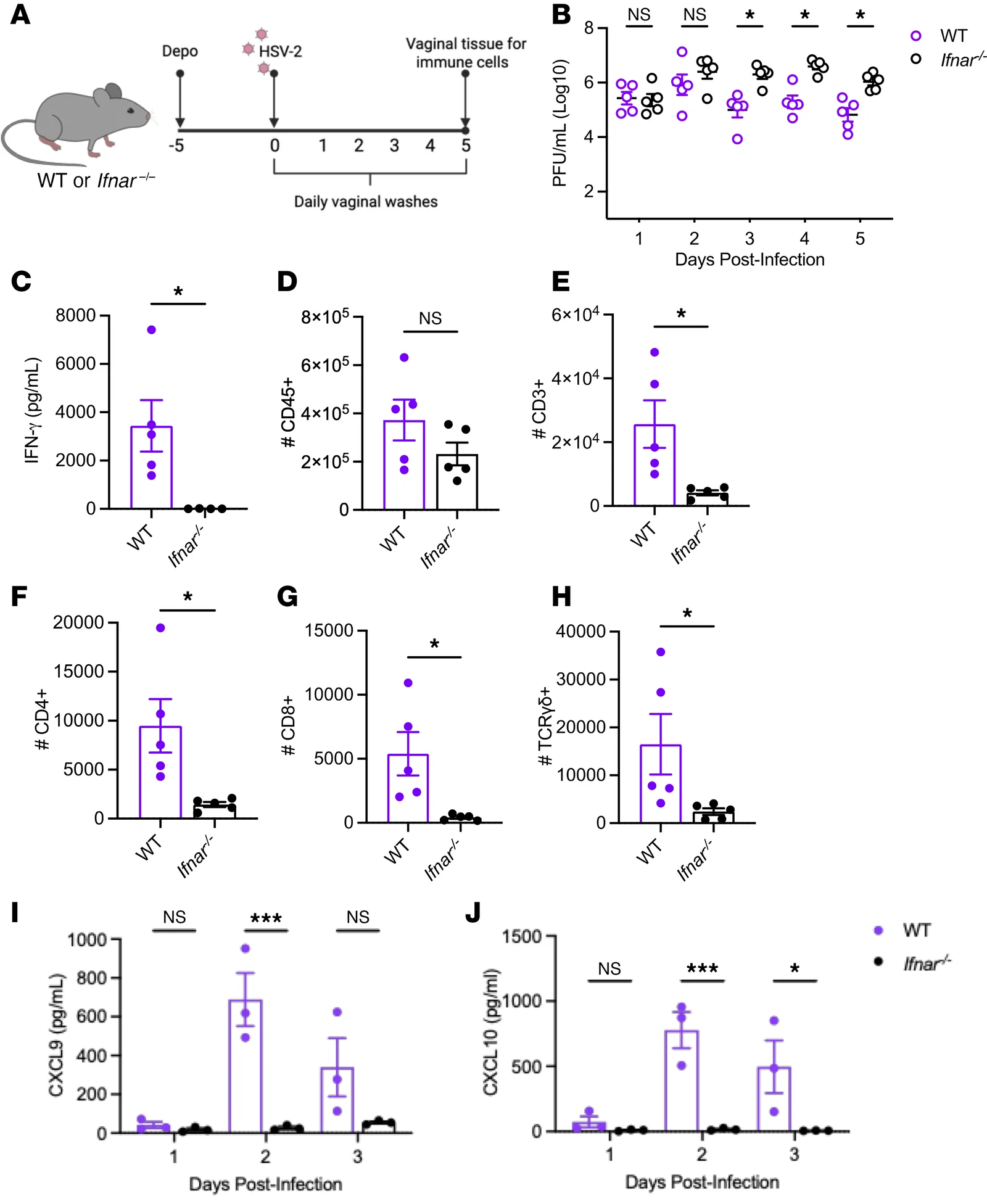
Absence of type I IFNs impairs adaptive immunity to HSV-2 infection. (**A**) WT and *Ifnar^–/–^* mice were infected with 10^4^ PFU HSV-2 333, and the vaginal tracts were collected 5 DPI (*n =* 5, 5). Data representative of 2 independent experiments. (**B**) Viral titers in the vaginal washes. (**C**) IFN-γ levels in vaginal washes 5 DPI. (**D**) Total number of CD45^+^ cells in the vaginal tract. (**E**) Total number of T cells (CD45^+^/Ly6G^–^/CD3^+^). (**F**) Total number of CD4^+^ T cells (CD45^+^/Ly6G^–^/CD3^+^/TCRβ^+^/TCRγδ^–^/CD8^–^/CD4^+^). (**G**) Total number of CD8^+^ T cells (CD45^+^/Ly6G^–^/CD3^+^/TCRβ^+^/TCRγδ^–^/CD8^+^/CD4^–^). (**H**) Total number of γδ T cells (CD45^+^/Ly6G^–^/CD3^+^/TCRβ^–^/TCRγδ^+^). (**I**) CXCL9 levels in vaginal washes. (**J**) CXCL10 levels in vaginal washes. *P < 0.05. ***P < 0.001 Statistical significance determined by 2-tailed Student’s *t* test (**C**–**H**) and repeated measures 2-way ANOVA with Šídák’s multiple comparisons test (**B**, **I**, and **J**).

**Figure 3:**
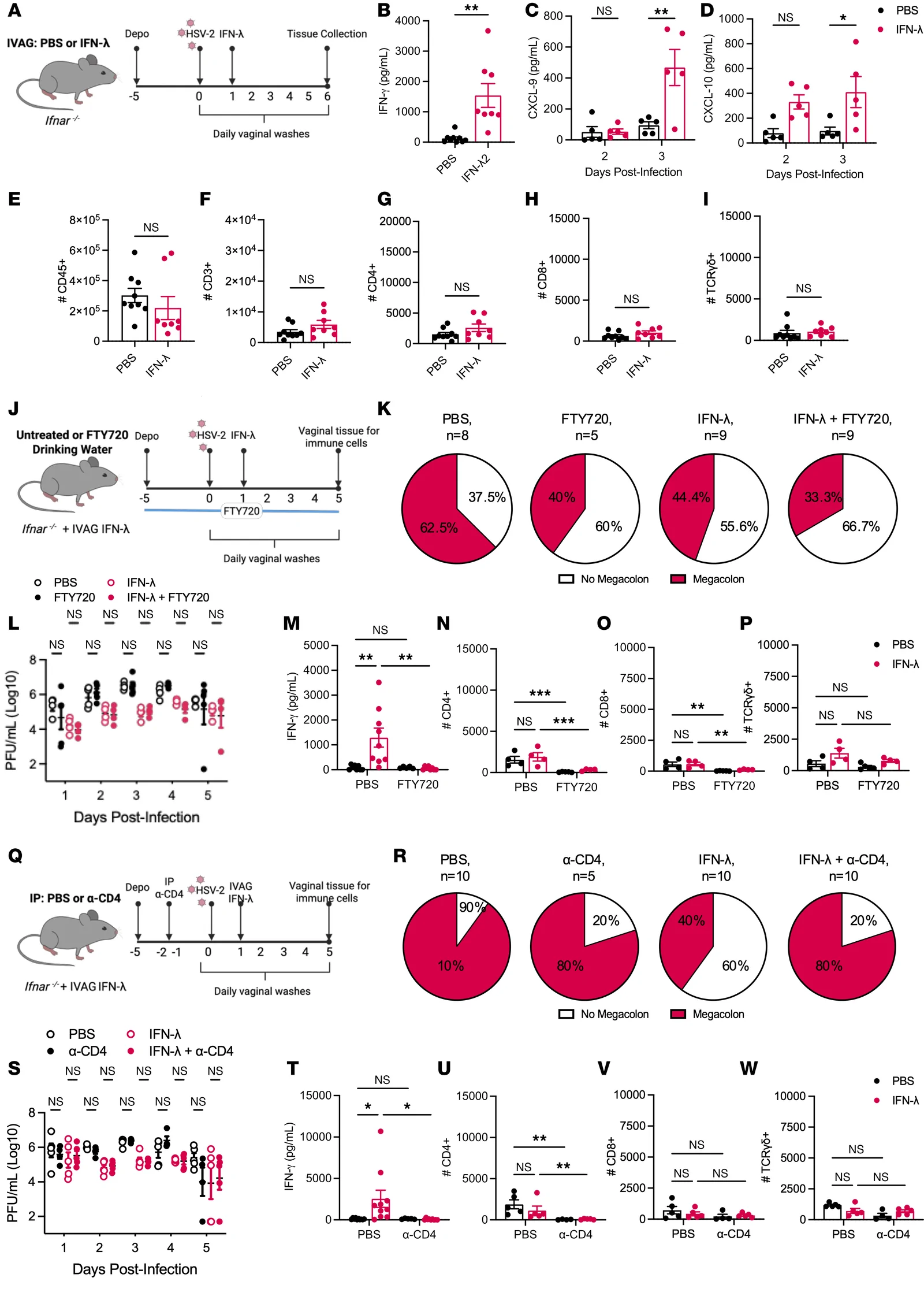
IFN-λ treatment boosts CD4^+^ T cell–mediated immunity to infection. (**A**–**I**) *Ifnar^–/–^* mice were infected with 10^4^ PFU HSV-2 333 and administered PBS or IFN-λ (*n* = 9, 8). Data representative of 2 pooled experiments, except **C**, which is representative of 2 experiments with similar results (*n* = 5, 5). (**B**) IFN-γ levels in vaginal washes 5 DPI. (**C**) CXCL9 levels. (**D**) CXCL10 levels. (**E**) Total number of CD45^+^ cells in the vaginal tissue. (**F**) Total number of T cells (CD45^+^/Ly6G^–^/CD3^+^). (**G**) Total number of CD4^+^ T cells (CD45^+^/Ly6G^–^/CD3^+^/TCRβ^+^/TCRγδ^–^/CD8^–^/CD4^+^). (**H**) Total number of CD8 T cells (CD45^+^/Ly6G^–^/CD3^+^/TCRβ^+^/TCRγδ^–^/CD8^+^/CD4^–^). (**I**) Total number of γδ^+^T cells (CD45^+^/Ly6G^–^/CD3^+^/TCRβ^–^/TCRγδ^+^). (**J**–**P**) *Ifnar^–/–^* mice were administered normal drinking water and PBS, FTY720 and PBS, normal drinking water and IFN-λ, or FTY720 and IFN-λ (*n* = 4, 5, 4, 4). Data representative of 2 independent experiments, except **K** and **M**, which are representative of 2 pooled experiments (*n =* 8, 5, 9, 9). (**K**) Rates of megacolon 5 DPI. (**L**) Viral titers. (**M**) IFN-γ levels. (**N**) Total number of CD4^+^ T cells. (**O**) Total number of CD8^+^ T cells. (**P**) Total number of TCRγδ^+^ cells 5 DPI. (**Q**–**W**) *Ifnar^–/–^* mice treated with IFN-λ 1 DPI intravaginally were administered PBS, α-CD4, IFN-λ, or α-CD4^+^IFN-λ (*n* = 5, 4, 5, 5). Data representative of 2 independent experiments, except **R** and **T**, which are representative of 2 pooled experiments (*n =* 10, 5, 10, 10). (**R**) Rates of megacolon 5 DPI. (**S**) Viral titers. (**T**) IFN-γ levels. (**U**) Total number of CD4^+^ T cells. (**V**) Total number of CD8^+^ T cells. (**W**) Total number of TCRγδ^+^ cells. *P < 0.05, **P < 0.01. Statistical significance determined by 2-tailed Student’s *t* test (**B** and **E**–**I**), repeated measures 2-way ANOVA with Šídák’s multiple comparisons test (**C**, **D**, **L**, and **S**), and ordinary 2-way ANOVA with Šídák’s multiple comparisons test (**M**–**P** and **T**–**W**).

**Figure 4:**
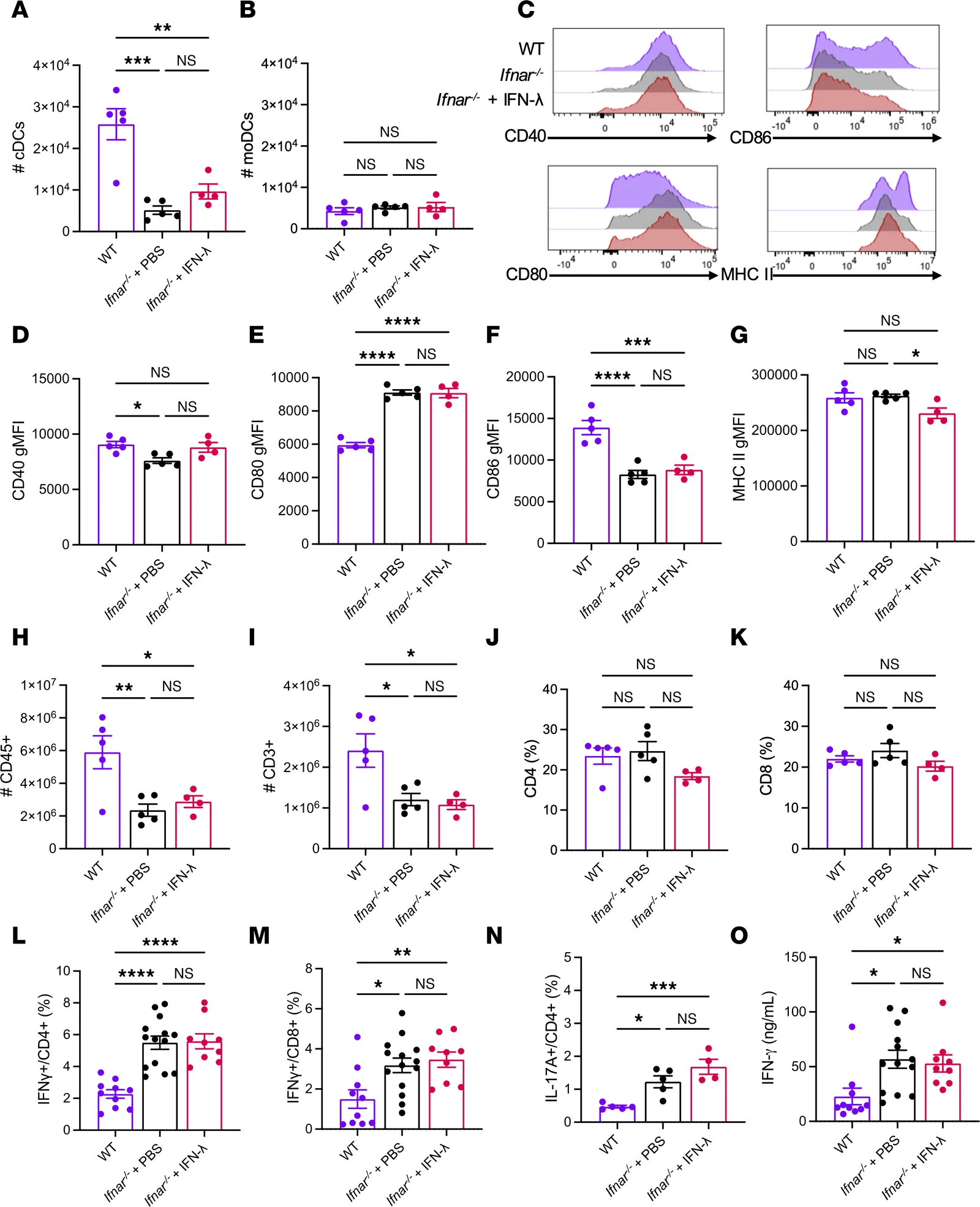
IFN-λ treatment does not alter T cell priming. (**A**–**O**) WT, *Ifnar^–/–^,* and *Ifnar^–/–^* administered IFN-λ 1 DPI (*n* = 5, 5, 4) were infected with 10^4^ PFU HSV-2 333. Data shown in **A**–**G** are representative of 2 independent experiments; **H**–**L** are representative of 3 independent experiments with similar results; and **M**–**O** are representative of 3 independent experiments pooled (*n* = 10, 14, 14). The dLN was collected 5 DPI. (**A**) Total number of cDCs (CD45^+^/B220^–^/CD3^–^/CD11c^+^/MHC II^+^/CD26^+^/CD64^–^). (**B**) Total number of moDCs (CD45^+^/B220^–^/CD3^–^/CD11c^+^/MHC II^+^/CD26^–^/CD64^+^). (**C**) Histograms for CD40, CD80, CD86, and MHC II for total DCs. (**D**) CD40 gMFI. (**E**) CD80 gMFI. (**F**) CD86 gMFI. (**G**) MHC II gMFI. (**H**) Total number of CD45^+^ cells. (**I**) Total number of CD3^+^ T cells (CD45^+^/CD3^+^). (**J**) Proportion of CD4^+^ T cells (CD45^+^/CD3^+^/TCRβ^+^/TCRγδ^–^/CD8^–^/CD4^+^) to total CD45^+^ cells. (**K**) Proportion of CD8^+^ T cells (CD45^+^/CD3^+^/TCRβ^+^/TCRγδ^–^/CD8^+^/CD4^–^) to total CD45^+^ cells. (**L**) Proportion of IFN-γ^+^CD4^+^ T cells to total CD4^+^ cells. (**M**) IFN-γ^+^CD8^+^ T cells to total CD8^+^ cells. (**N**) Proportion of **L**. IL-17A^+^CD4^+^ T cells to total CD4^+^ cells. (**O**) IFN-γ levels in the supernatants. *P < 0.05, **P < 0.01. ***P < 0.001. Statistical significance was determined by 1-way ANOVA with Tukey’s multiple comparisons test. gMFI, geometric mean fluorescence intensity.

**Figure 5:**
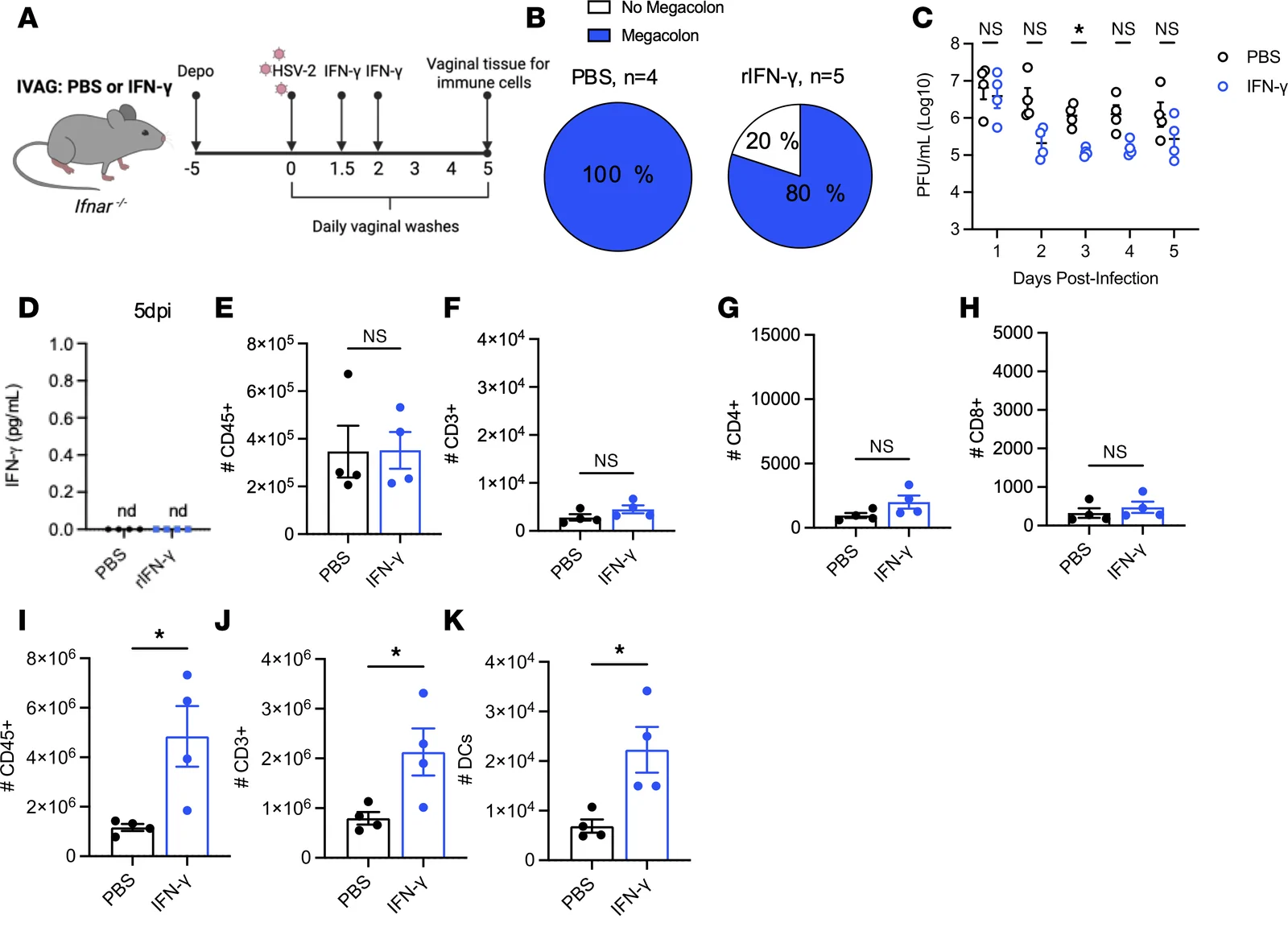
Innate IFN-γ does not influence local T cell responses. (**A**) *Ifnar^–/–^* mice were infected with 10^4^ PFU HSV-2 333 and administered PBS 1.5 and 2 DPI intravaginally. Data shown are representative of 1 independent experiment. (**B**) Rates of megacolon 5 DPI (*n* = 4, 5). (**C**) Viral titers in vaginal washes (*n* = 4, 4). (**D**) IFN-γ in the vaginal washes 5 DPI (*n* = 4, 4). (**E**) Total number of CD45^+^ cells in the vaginal tissue 5 DPI (*n* = 4, 4). (**F**) Total number of CD3^+^ T cells (CD45^+^/Ly6G^–^/CD3^+^) in vaginal tissue. (**G**) Total number of CD4^+^ T cells (CD45^+^/Ly6G^–^/CD3^+^/TCRβ^+^/TCRγδ^–^/CD8^–^/CD4^+^) in the vaginal tissue. (**H**) Total number of CD8^+^ T cells (CD45^+^/Ly6G^–^/CD3^+^/TCRβ^+^/ TCRγδ^–^/CD8^+^/CD4^–^) in the vaginal tissue. (**I**) Total number of CD45^+^ cells in the dLN 5 DPI. (**J**) Total number of CD3^+^ T cells (CD45^+^/CD3^+^) in the dLN. (**K**) Total number of DCs (CD45^+^/B220^–^/CD3^–^/CD11c^+^/MHCII^+^) in the dLN. *P < 0.05. Statistical significance determined by 2-way ANOVA with Šídák’s multiple comparisons test (**C**) or 2-tailed Student’s *t* test (**D**–**K**).

**Figure 6:**
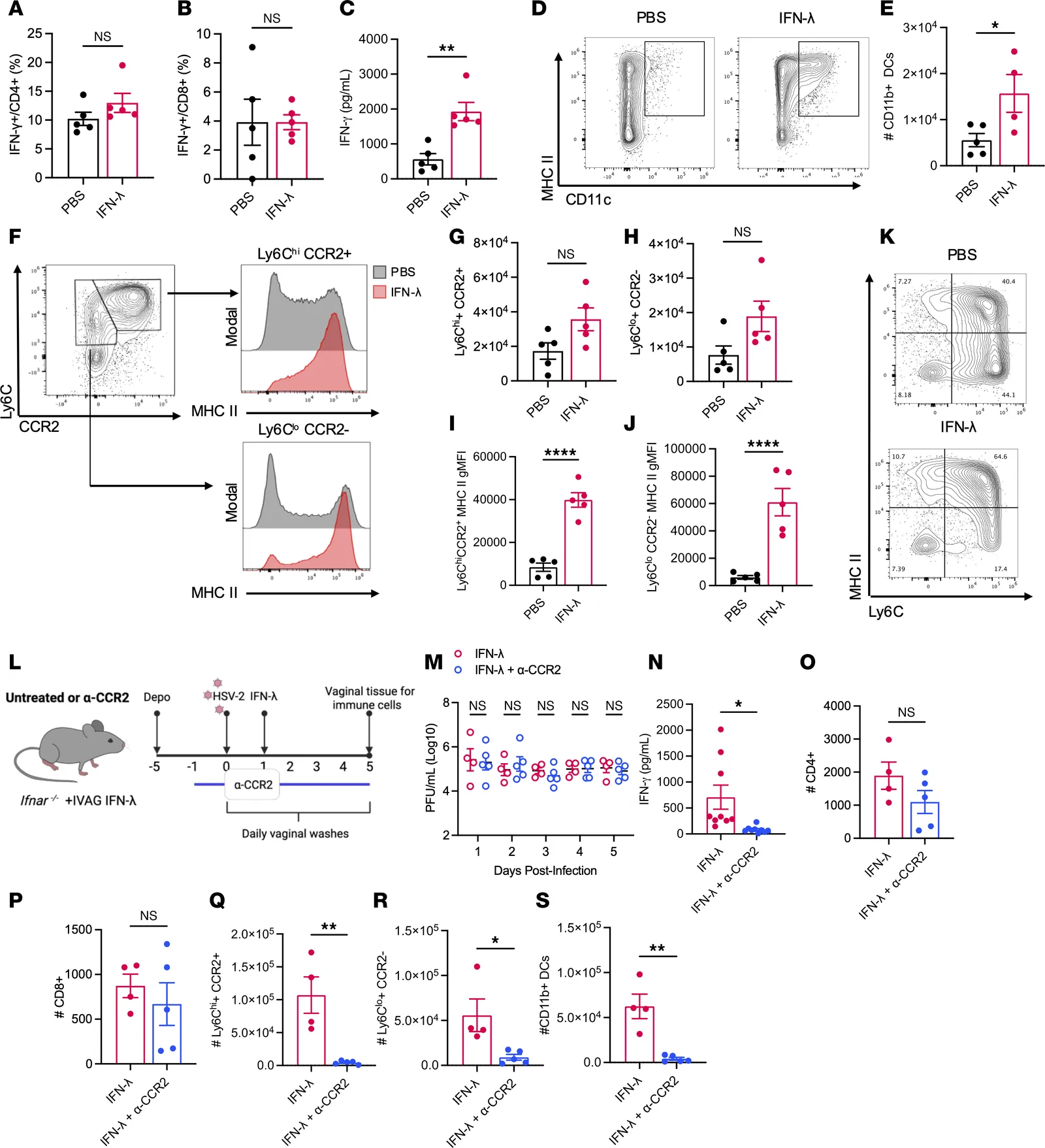
IFN-λ boosts CD4^+^ T cell function through CCR2^+^ inflammatory monocytes. (**A**–**C**) *Ifnar^–/–^* mice were infected with 10^4^ PFU HSV-2 333 and administered PBS or IFN-λ 1 DPI (*n* = 5, 5). Data representative of 2 independent experiments. (**A**) Total vaginal cells were collected 5 DPI and stimulated with HSV-2 lysate for 24 hours. Proportion of IFN-γ^+^CD4^+^ T cells to total CD4^+^ cells. (**B**) Proportion of IFN-γ^+^CD8^+^ T cells to total CD8^+^ cells. (**C**) Total IFN-γ levels in supernatants. (**D**) Representative flow plots of CD11b^+^ DCs (CD45^+^/Ly6G^–^/CD3^–^/B220^–^/CD11b^+^/CD11c^+^/MHCII^+^) from unstimulated vaginal cells. (**E**) Total number of CD11b^+^ DCs 5 DPI (*n* = 5, 4). (**F**) Representative flow plots for Ly6C^hi^CCR2^+^ (CD45^+^/Ly6G^–^/CD3^–^/B220^–^/CD11b^+^/Ly6C^hi^/CCR2^+^) and Ly6C^lo^CCR2^–^ (CD45^+^/Ly6G^–^/CD3^–^/B220^–^/CD11b^+^/Ly6C^lo^/CCR2^–^) cells to assess MHC II gMFI. (**G**) Total number of Ly6C^hi^CCR2^+^ cells (*n* = 5, 4). (**H**) Total number of Ly6C^lo^CCR2^–^ cells. (**I**) MHC gMFI of Ly6C^hi^CCR2^+^ cells. (**J**) MHC gMFI of Ly6C^lo^CCR2^–^ cells. (**K**) Representative flow plots for comparing Ly6C and MHC II expression in CD11b^+^ cells. (**L**) *Ifnar^–/–^* mice infected with HSV-2 333 and administered IFN-λ were administered PBS or α-CCR2 daily (*n* = 4, 5). Data shown are representative of 2 independent experiments with similar results, except **N**, which is representative of 2 pooled experiments (*n* = 9, 9). (**M**) Viral titers. (**N**) IFN-γ levels in vaginal washes 5 DPI. (**O**) Total number of CD4^+^ T cells. (**P**) Total number of CD8^+^ T cells. (**Q**) Total number of Ly6C^hi^CCR2^+^ cells. (**R**) Total number of Ly6C^lo^CCR2^–^ cells. (**S**) Total number of CD11b^+^ DC cells. *P < 0.05, **P < 0.01 ***P < 0.0001, ****P < 0.00001. Statistical significance determined by repeated 2-way ANOVA with Šídák’s multiple comparisons test (**M**) or 2-tailed Student’s *t* test (**A**–**C**, **E**, **G**–**J**, and **N**–**S**). gMFI, geometric mean fluorescence intensity.

## Version 1 10/15/2026

Electronic publication
