# Supplementary material for: Interferon-λ inhibits immunopathology and boosts T cell–mediated immunity in a mouse model of mucosal viral infection
Source: J Clin Invest. 2026 Oct 15;136(20):e198863. doi: 10.1172/JCI198863 (PMC13668623; doi:10.1172/JCI198863)
Supplement: Supplementary file 1 [file jci-136-198863-s223.pdf]

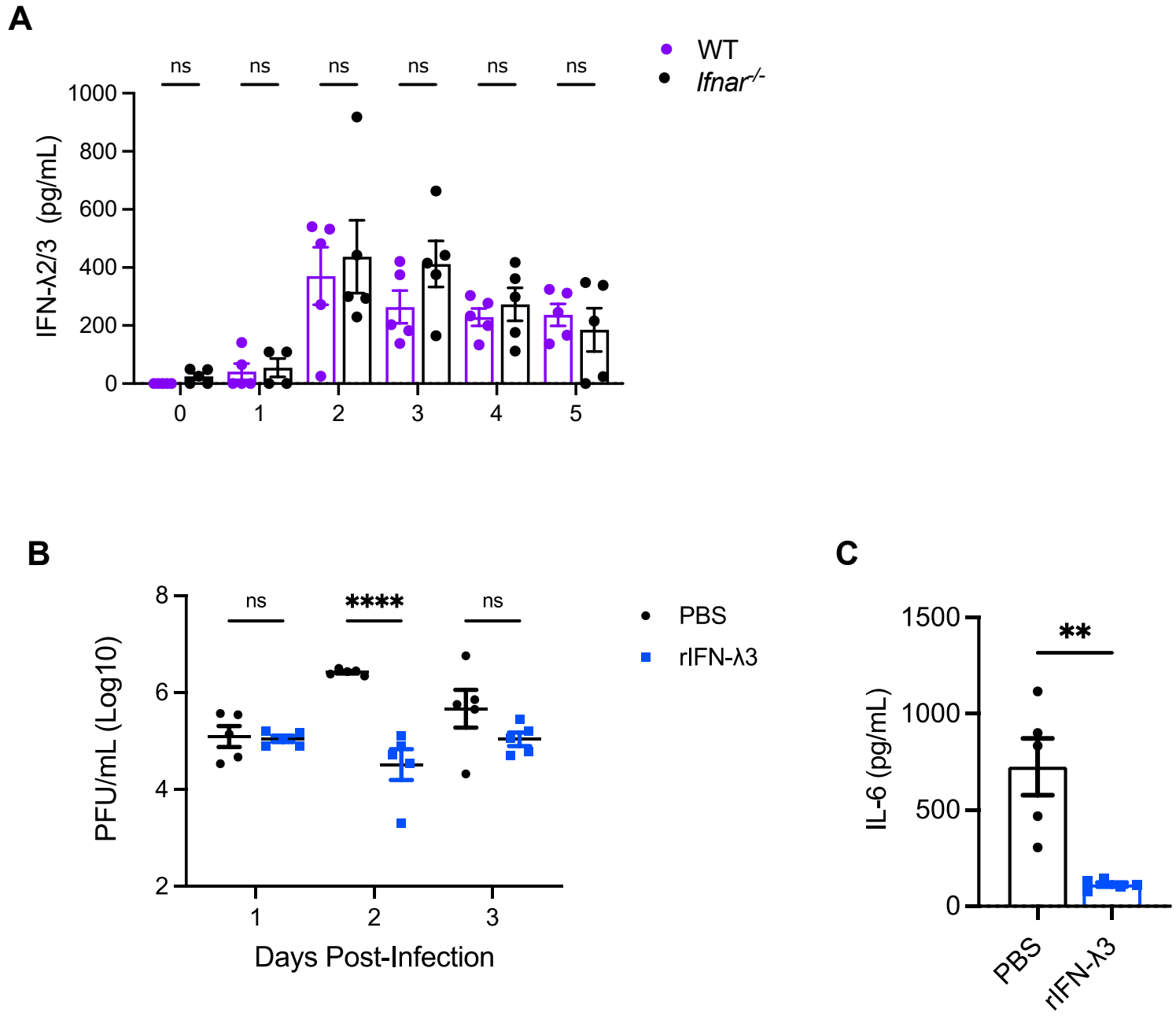

Fig. S1. IFN-λ3 treatment inhibits IL-6-mediated pathology and rescues lymphocyte-derived IFN-γ. A. WT (*n* = 5) and *Ifnar*$^{-/-}$ (*n* = 5) mice were infected with 10^4 PFU HSV-2 333, and IFN-λ 2/3 levels quantified in the vaginal washes. B-C *Ifnar*$^{-/-}$ mice were infected with 10^4 PFU HSV-2 strain 333 and administered PBS (*n* = 5) or 5 µg IFN-λ3 (*n* = 5) 1 DPI. B. Viral titres in vaginal washes. C. IL-6 levels in vaginal washes 3 DPI. ns, not significant. ** $P < 0.01$, **** $P < 0.0001$. Statistical significance determined by two-way ANOVA with Sidak's multiple comparisons test (A, B) or two-tailed student *t*-test (C).

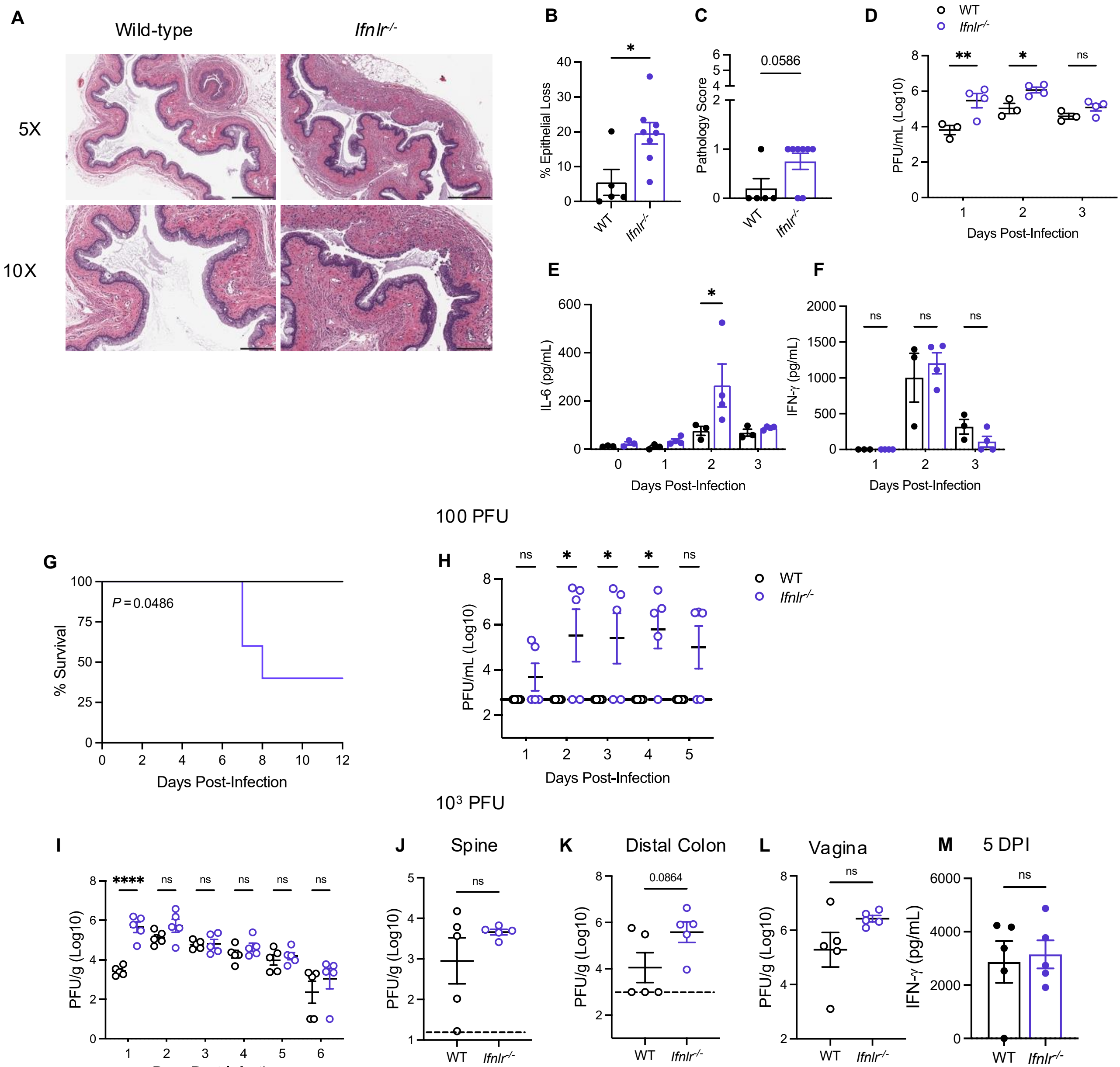

Fig. S2. Loss of *Ifnlr* impairs early antiviral immune responses. A-F. WT and *Ifnlr*$^{-/-}$ mice were infected with 10^4 PFU HSV-2 333. A. Representative H&E staining. B. % epithelial loss and C. pathology score of WT (*n* = 5) and *Ifnlr1*$^{-/-}$ (*n* = 8) mice. D. Viral titres in vaginal washes of WT (*n* = 3) and *Ifnlr1*$^{-/-}$ (*n* = 4) mice E. IL-6 levels in vaginal washes. F. IFN-γ levels in vaginal washes. G-H. WT (*n* = 5) and *Ifnlr*$^{-/-}$ (*n* = 5) mice were infected with 100 PFU HSV-2 strain 333. G, Survival Curve. H. Viral titers in the vaginal washes. I-J. WT (*n* = 5) and *Ifnlr*$^{-/-}$ (*n* = 5) mice were infected with 10^3 PFU HSV-2 strain 333. I. Viral titers in vaginal washes. J. Viral titers in spine 6 DPI. K. Viral titers in distal colon 6 DPI. L. Viral titers in whole vaginal tissue 6 DPI. M. IFN-γ levels in vaginal washes 5 DPI. ns, not significant. * $P < 0.05$, ** $P < 0.01$, **** $P < 0.0001$. Statistical significance determined by Kaplan-Meier survival analysis (G) two-way ANOVA with Sidak's multiple comparisons test (D, E, F, H, I) or two-tailed Student's *t*-test (B, C, J, K, L, M).

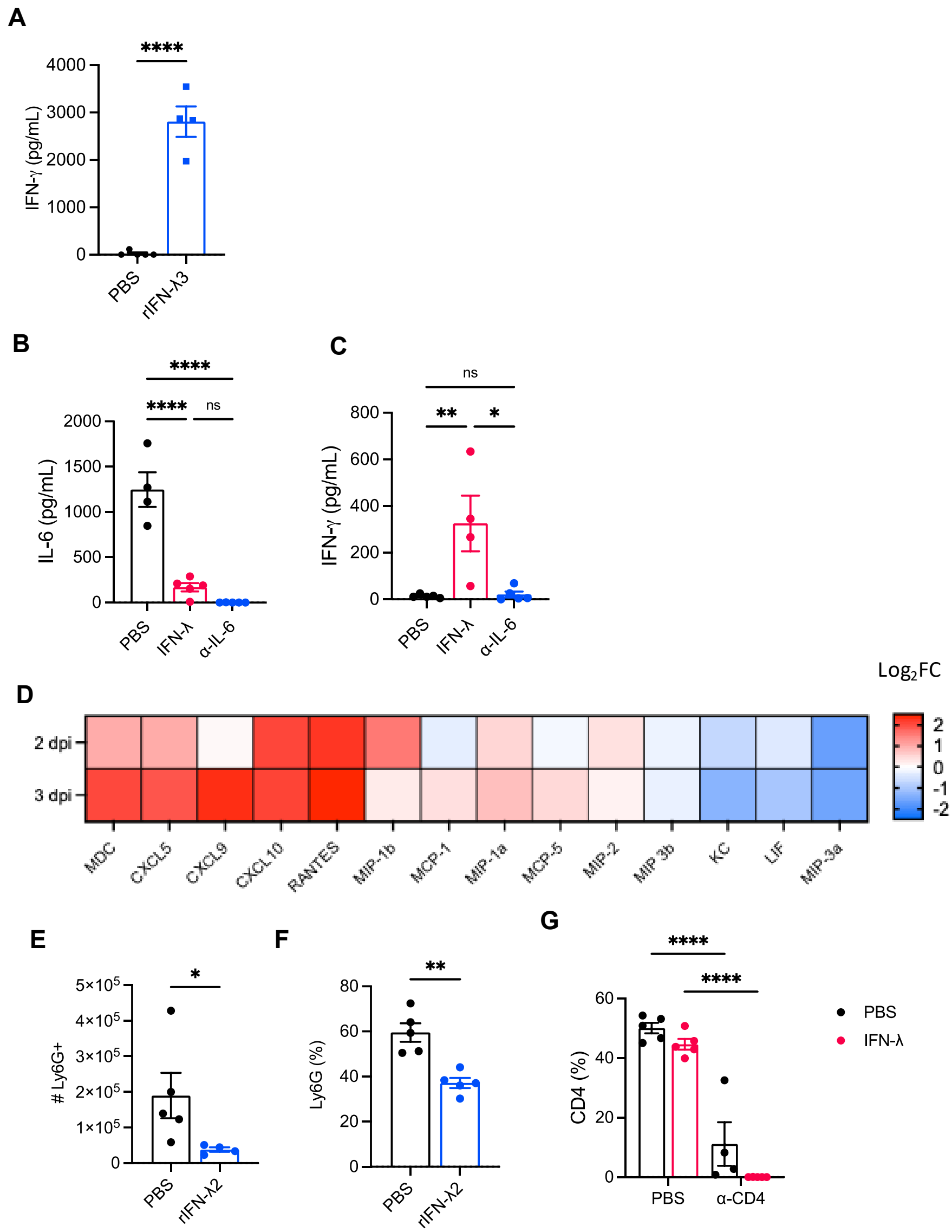

Fig. S3. IFN-λ induces late-stage IFN-γ production independent of increasing T cell recruitment. A. *Ifnar-/-* mice were administered PBS (*n* = 5) or IFN-λ (*n* = 4), and IFN-γ levels were measured 5 DPI in the vaginal washes. B-C, *Ifnar-/-* mice were administered PBS (*n* = 4), IFN-λ (*n* = 4), or α-IL-6 (*n* = 5) during HSV-2 infection B. IL-6 levels in vaginal washes 3 DPI. C. IFN-γ levels in vaginal washes 5 DPI. D-F. *Ifnar-/-* mice were infected with 104 PFU HSV-2 strain 333 and administered PBS (*n* = 5) or IFN-λ (*n* = 5). D. Log2FC of cytokine production 2 and 3 DPI, as fold change over PBS-treated mice. E. Total number of neutrophils (CD45+/Ly6G+). F. Proportion of neutrophils to total CD45+ cells. G. *Ifnar-/-* mice treated with IFN-λ 1 DPI intravaginally were administered PBS (*n* = 5), α-CD4 (*n* = 5), IFN-λ (*n* = 4), or IFN-λ + α-CD4 *(n* = 5), 1 and 2 days before infection. Proportion of CD4+ T cells (CD45+/CD3+/TCRβ+/TCRγδ-/CD8-/CD4+) to total CD45+ cells in the dLN 5 DPI. ns, not significant. * $P < 0.05$, *** $P < 0.001$, **** $P < 0.0001$. Statistical significance determined by two-way ANOVA with Sidak's multiple comparisons test (G), one-way ANOVA with Sidak's multiple comparisons test (B, C), with or two-tailed student *t*-test (E, F).

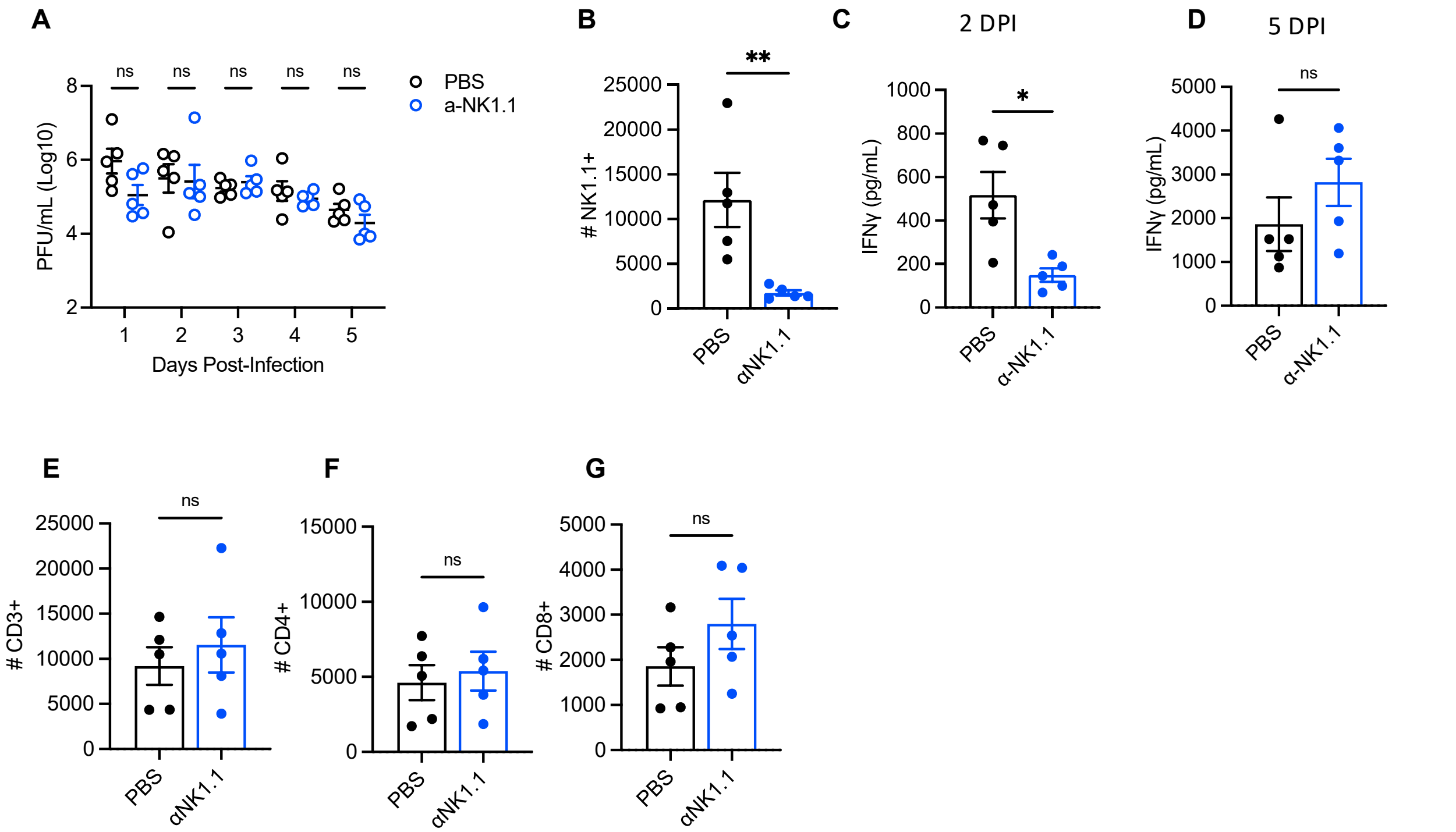

Fig. S4. NK cell depletion does not impact T cell function. A-J. WT mice were infected with 104 PFU HSV-2 strain 333 and administered PBS (*n* = 5) or α-NK1.1 (*n* = 5). Tissues were collected 5 DPI. A. Viral titres. B. Total number of NK cells in the vaginal tissue 5 DPI. C. IFN-γ in the vaginal washes 2 DPI. D. IFN-γ in the vaginal washes 5 DPI. E-H. Total number of CD45 +cells in the vaginal tissue E. Total number of T cells (CD45+/Ly6G-/CD3+) F. Total number of CD4+ T cells (CD45+/Ly6G-/CD3+/TCRβ+/CD4+/CD8-) G. Total number of CD8+ T cells (CD45+/Ly6G-/CD3+/TCRβ+/CD4-/CD8+). ns, not significant. ** $P < 0.01$. Statistical significance determined by two-way ANOVA with Sidak's multiple comparisons test (A), two-tailed student *t*-test (B-G).

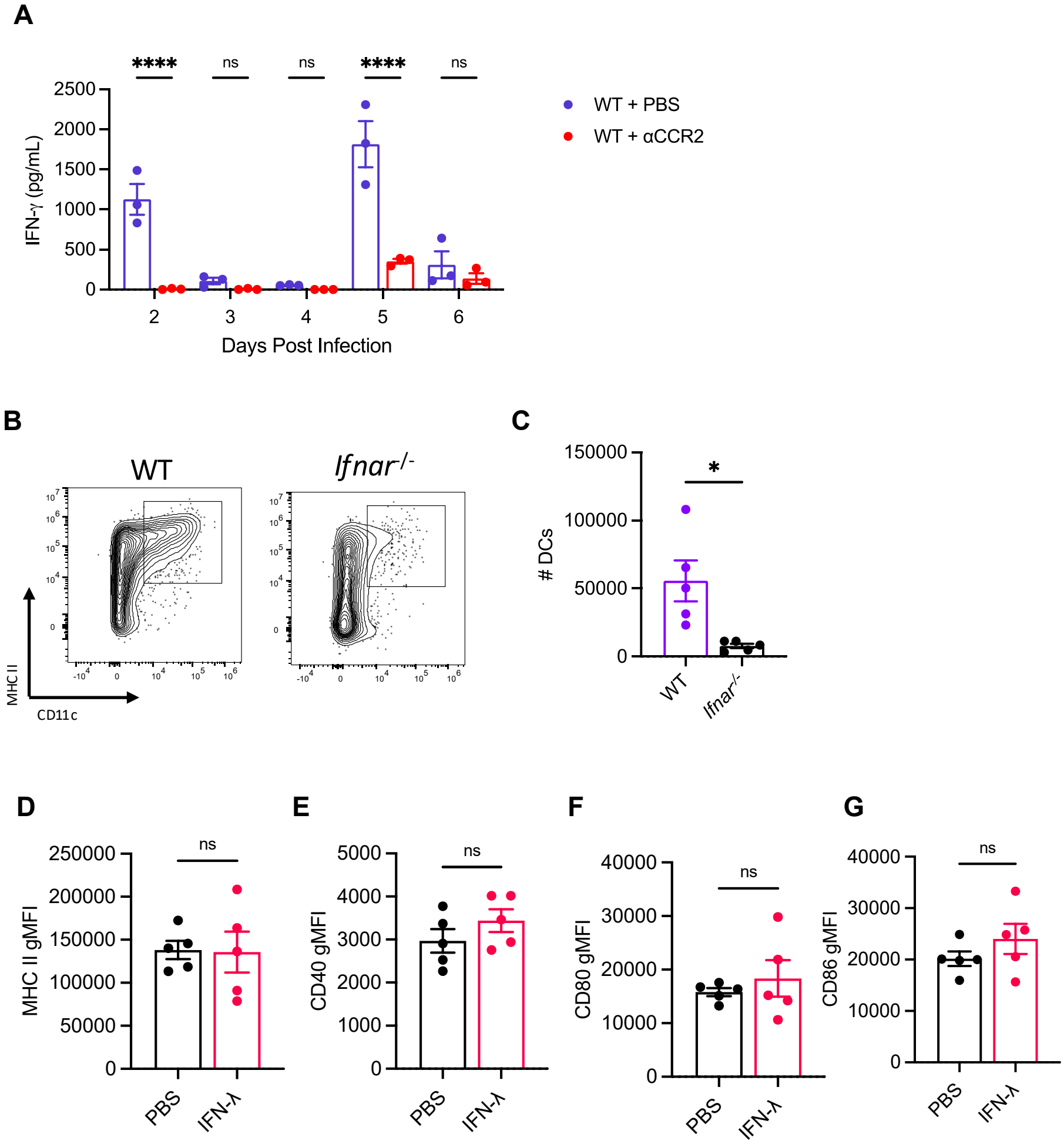

Fig S5. CCR2+ cells are necessary for CD4+ T cell IFN-γ production. A. WT mice were administered PBS (*n* = 3) or α- CCR2 (*n* = 3), and IFNγ levels were measured 2-6 DPI. B WT (*n* = 5) and *Ifnar-/-* mice (*n* = 5) were infected with 104 PFU HSV-2 and vaginal tissue was collected 5 DPI. Representative flow plots for CD11b+ DCs. C. Total number of CD11b+ DCs (CD45+/Ly6G-/B220-/CD3-/CD11b+/CD11c+/MHC II+) in the vaginal tissue. D. *Ifnar-/-* mice (*n* = 5) were infected with 104 PFU HSV-2 and treated with PBS(*n* = 5) or IFN-λ (*n* = 5) 1 DPI intravaginally. Vaginal tissue was collected 5 DPI. E. MHC II gMFI of CD11b+ DCs treated mice. F. CD40 II gMFI of CD11b+ DCs. G. CD80 gMFI of CD11b+ DCs. H. CD80 gMFI of CD11b+ DCs. ns, not significant. * *P* < 0.05, **** *P* < 0.001. Statistical significance determined by two-way ANOVA with Sidak's multiple comparisons test (A), two-tailed student *t*-test (C-G).

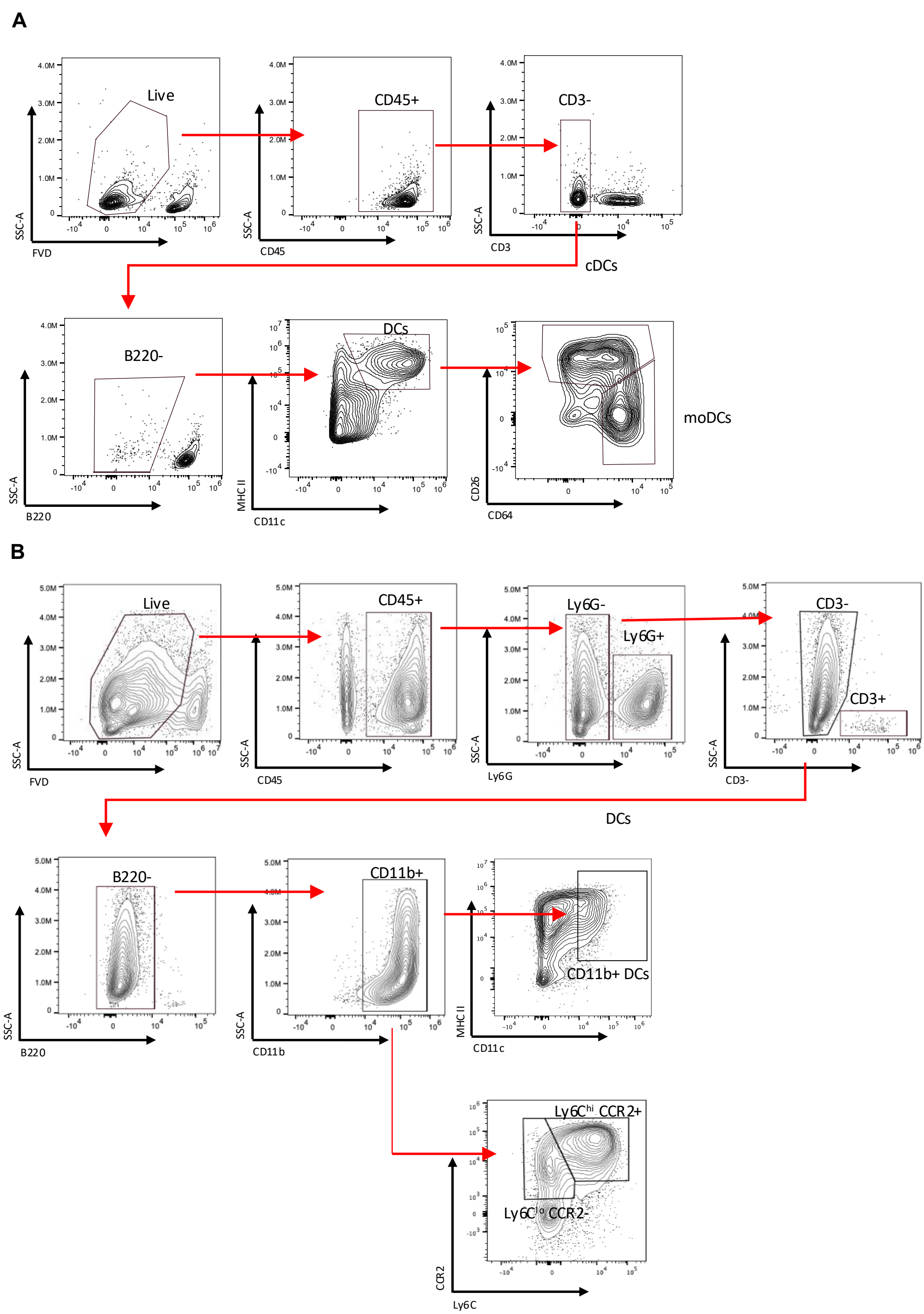

Fig S6. Gating Strategy for DCs in the dLN and vaginal tract. A. Gating strategy for DCs in the LN. B. Gating strategy for DCs in the vaginal tract
